# Investigating underlying molecular mechanisms, signaling pathways, emerging therapeutic approaches in pancreatic cancer

**DOI:** 10.3389/fonc.2024.1427802

**Published:** 2024-07-17

**Authors:** Mohd Mustafa, Kashif Abbas, Mudassir Alam, Safia Habib, Zulfareen, Gulam Mustafa Hasan, Sidra Islam, Anas Shamsi, Imtaiyaz Hassan

**Affiliations:** ^1^ Department of Biochemistry, J.N. Medical College, Faculty of Medicine, Aligarh Muslim University, Aligarh, India; ^2^ Department of Zoology, Faculty of Life Sciences, Aligarh Muslim University, Aligarh, India; ^3^ Centre for Interdisciplinary Research in Basic Sciences, Jamia Millia Islamia, New Delhi, India; ^4^ Department of Basic Medical Science, College of Medicine, Prince Sattam Bin Abdulaziz University, Al-Kharj, Saudi Arabia; ^5^ Department of Inflammation & Immunity, Lerner Research Institute, Cleveland Clinic, Cleveland, OH, United States; ^6^ Center of Medical and Bio-Allied Health Sciences Research (CMBHSR), Ajman University, Ajman, United Arab Emirates

**Keywords:** pancreatic adenocarcinoma, therapeutic advancements, tumor microenvironment, neoadjuvant therapies, immunotherapy, clinical manifestations

## Abstract

Pancreatic adenocarcinoma, a clinically challenging malignancy constitutes a significant contributor to cancer-related mortality, characterized by an inherently poor prognosis. This review aims to provide a comprehensive understanding of pancreatic adenocarcinoma by examining its multifaceted etiologies, including genetic mutations and environmental factors. The review explains the complex molecular mechanisms underlying its pathogenesis and summarizes current therapeutic strategies, including surgery, chemotherapy, and emerging modalities such as immunotherapy. Critical molecular pathways driving pancreatic cancer development, including KRAS, Notch, and Hedgehog, are discussed. Current therapeutic strategies, including surgery, chemotherapy, and radiation, are discussed, with an emphasis on their limitations, particularly in terms of postoperative relapse. Promising research areas, including liquid biopsies, personalized medicine, and gene editing, are explored, demonstrating the significant potential for enhancing diagnosis and treatment. While immunotherapy presents promising prospects, it faces challenges related to immune evasion mechanisms. Emerging research directions, encompassing liquid biopsies, personalized medicine, CRISPR/Cas9 genome editing, and computational intelligence applications, hold promise for refining diagnostic approaches and therapeutic interventions. By integrating insights from genetic, molecular, and clinical research, innovative strategies that improve patient outcomes can be developed. Ongoing research in these emerging fields holds significant promise for advancing the diagnosis and treatment of this formidable malignancy.

## Introduction

Pancreatic cancer, characterized by its aggressive behavior, a tendency for late-stage identification, and limited therapeutic options, poses a significant challenge in the advancing field of oncology (1). The tumor microenvironment (TME) comprises a dynamic amalgamation of immune cells, extracellular matrix, and stromal cells, significantly influencing the disease trajectory and complicating treatment resistance (2). Epithelial-mesenchymal transition (EMT) promotes cancer cell invasion and migratory capabilities, intensifying cancer cell complexity. The immune evasion mechanisms employed by pancreatic cancer cells pose a formidable barrier to effectively utilizing immunotherapy, necessitating innovative solutions (3).

Critical signaling pathways govern the crucible of cellular life. Furthermore, persistent activation of the KRAS pathway is a hallmark feature of uncontrolled cell survival and proliferation (4). The Hedgehog and Notch pathways contribute to the resilience of cancer stem cells, increasing their resistance to treatment (5). Dysregulation of the PI3K/AKT/mTOR pathway promotes increased cellular growth and survival (6). The Wnt/β-catenin signaling pathway activates tumor growth, further complicating the battle against pancreatic cancer (7). Surgical excision remains the primary curative option for early-stage patients (8). In more advanced stages, accepted standard-of-care options include chemotherapy regimens such as gemcitabine, FOLFIRINOX, and nab-paclitaxel (9). Localized tumors may undergo radiation treatment to eliminate or reduce their presence. Several targeted therapies, particularly PARP inhibitors, are currently under rigorous investigation to treat pancreatic cancer (10). Immunotherapy involving checkpoint inhibitors and vaccines holds promise for enhancing the immune system’s response to pancreatic cancer (11).

Liquid biopsies are being explored as noninvasive diagnostic tools for the primary detection of pancreatic cancer, potentially enabling intervention at a more treatable stage (12). Personalized medicine approaches, tailored to individuals’ genetic and molecular profiles, are poised to optimize therapeutic strategies, providing a specialized toolkit against this resilient adversary. The application of CRISPR/Cas9 genome editing tools for the exploration and potential correction of genetic mutations is actively being explored, revealing the possibility of addressing the illness at its molecular origins. Artificial intelligence has been harnessed to expedite the early identification and prediction of therapeutic responses in pancreatic cancer patients, demonstrating the power of technology to treat pancreatic carcinoma (13).

The primary objectives of this review are to provide a comprehensive overview of the current molecular and genetic landscape of PDAC, including an in-depth examination of key molecular pathways such as KRAS, Notch, and Hedgehog, and their roles in the pathogenesis and progression of the disease. Additionally, this review aims to critically analyse existing therapeutic strategies and their limitations, offering a thorough evaluation of conventional treatments like surgery, chemotherapy, and radiation, as well as emerging therapies such as immunotherapy and targeted molecular treatments. The challenges associated with these treatments, particularly issues related to drug resistance and the tumor microenvironment, will be highlighted. Furthermore, the review seeks to highlight the potential of emerging diagnostic and therapeutic technologies. This involves exploring novel approaches such as liquid biopsies for early detection, personalized medicine based on genomic and transcriptomic profiling, and the application of CRISPR/Cas9 gene editing technology. The review aims to identify key challenges and propose future research directions. This includes recognizing major obstacles in the treatment and management of PDAC, such as the tumor micro-environment and immune evasion mechanisms. The review will propose future research directions aimed at overcoming these challenges, thereby facilitating the way for more effective diagnostic and therapeutic strategies.

## Mechanistic insights

**Figure 1:**
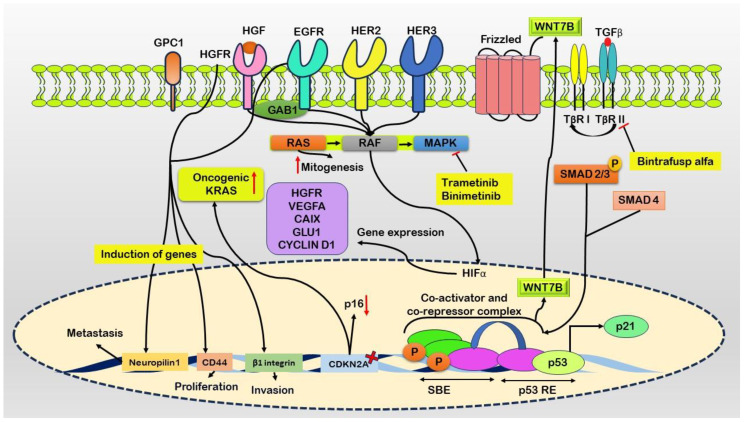
Schematic representation of molecular events driving pancreatic cancer progression. Ligand binding to the Epidermal Growth Factor Receptor (EGFR) initiates heterodimerization with ERRB2/HER2 and HER3. Co-occurrence of oncogenic KRAS mutations and elevated ligand expression enhances downstream signaling. Growth factor receptor-bound protein 1 (GAB1) augments activation of EGFR and Hepatocyte Growth Factor Receptor (HGFR). Glypican-1 (GPC1) maintains signaling pathways promoting mitogenesis, invasion, and metastasis via canonical RAS, RAF, MAPK, STAT3, PI3K, and AKT pathways. Fibroblast growth factor receptor substrate 2 (FRS2) is crucial for downstream signaling from FGFR1, triggering the Ras cascade. MAPK translocates to the nucleus to regulate transcription, including Hypoxia-Inducible Factor 1 (HIF1) induction. Dysfunctional retinoblastoma protein 1 (RB1) exacerbates mitogenic signaling and may convert Transforming Growth Factor-beta (TGFβ) into a direct mitogen through non-canonical MAPK and PI3K pathways. TGFβ-mediated activation of WNT7B occurs via a SMAD4-dependent mechanism. Elevated expression of growth factor receptors, such as HGFR and EGFR, induces genes like Neuropilin1, CD44, and β1 integrin, contributing to metastasis, proliferation, and invasion. Inhibitors such as Trametinib and Binimetinib target the MAPK pathway, while Bintrafusp alfa binds to TGFβ, leading to its blockade.

Pancreatic intraepithelial neoplasia (PanIN), a frequently encountered preneoplastic lesion, serves as the primary instigator of pancreatic cancer (14). Furthermore, more sophisticated precursor abnormalities, such as mucinous cystic neoplasms and intraductal papillary mucinous neoplasms (IPMNs), actively contribute to the development of this condition (15). The progression of pancreatic cancer involves complex molecular and cellular processes, with distorted autocrine and paracrine signaling pathways playing crucial roles in fostering the growth, migration, invasion, and metastasis of cancer cells. Critical factors, including transforming growth factor-α (TGFα) (16), insulin-like growth factor 1 (IGF1) (17), fibroblast growth factors (FGFs) (18), and hepatocyte growth factor (HGF) (19), along with their corresponding tyrosine kinase receptors, such as epidermal growth factor receptor (EGFR) (20), receptor tyrosine-protein kinase erbB-2 (ERBB2/HER2) (21), HER3 (22), the IGF1 receptor (IGF1R) (23), FGF receptors (FGFRs) (24), and the HGF receptor (HGFR/MET) (25), trigger several pathways contributing to cell growth (**Figure 1**).

Initial activation ensues upon ligand binding, activating EGFR and forming heterodimers with the receptor tyrosine-protein kinase erbB-2 (ERRB2/HER2) and HER3 (26). The coexistence of oncogenic KRAS and heightened ligand expression synergistically amplifies downstream signaling cascades (27). Docking protein growth factor receptor-bound protein 2 (GRB2)-associated binding protein 1 (GAB1) further enhances the activation of both EGFR and the hepatocyte growth factor (HGF) receptor (HGFR) (28). Prolonged signaling is sustained through the overexpression of heparan sulfate proteoglycan glypican 1 (GPC1), which promotes mitogenesis, invasion, and metastasis via canonical RAS, RAF, mitogen-activated protein kinase (MAPK), and other pathways, including signal transducer and activator of transcription 3 (STAT3), phosphatidylinositol 3-kinase (PI3K), and AKT pro-survival signaling (29).

The crucial adaptor protein fibroblast growth factor receptor substrate 2 (FRS2) is indispensable for downstream signaling from fibroblast growth factor receptor 1 (FGFR1), thereby activating the Ras signaling cascade (30). Subsequently, MAPK translocate to the nucleus, where it coordinates transcriptional activities, including the induction of hypoxia-inducible transcription factor 1 (HIF1) (31). Concurrently, dysfunctional retinoblastoma-associated protein (RB1) intensifies mitogenic signaling, potentially converting transforming growth factor-beta (TGFβ) into a direct mitogen through noncanonical pathways (MAPK and PI3K). Additionally, TGFβ-mediated activation of WNT7B is facilitated via a SMAD4-dependent mechanism (32).

Pancreatic cancer involves pathways that promote cell survival and inhibit apoptosis, particularly pathways involving AKT, NF-κB, and STAT3 (33). The reactivation of developmental genes such as *WNT*, *SHH*, and *NOTCH* occurs in certain pancreatic tumors (34). Aberrant crosstalk pathways and multiple nodes further compound the complicated signaling network of pancreatic cancer. For instance, heightened action of HGFR and EGFR leads to the induction of neuropilin1, CD44, and β1 integrin, contributing to an abnormal signaling node (35). The formation of heterodimers between HGFR and EGFR aggravates this complexity (36).

Simultaneously, these molecular changes occur with the deletion of CDKN2A, which is responsible for encoding the tumor suppressor p16, and the activation of oncogenic KRAS (37). Metabolic irregularities and a diminished response to growth-inhibitory pathways mark pancreatic cancer. One example of a lack of negative growth limitations is dysregulated TGFβ signaling, which is usually a tumor suppressor but paradoxically promotes tumor development in pancreatic cancer. TGFβ exerts paracrine effects within the tumor microenvironment, augmenting growth and metastatic processes (38).

Moreover, pancreatic cancer cell proliferation is directly stimulated by TGFβ through noncanonical signaling pathways. These pathways involve the phosphorylation of MAPK, the proto-oncogene tyrosine-protein kinase Src (SRC), AKT phosphorylation, and canonical SMAD4-dependent mechanisms that lead to the upregulation of WNT7B expression (39). Trametinib and binimetinib function as inhibitors of the MAPK pathway.

## Signaling pathways in pancreatic cancer

### K-Ras

**Figure 2:**
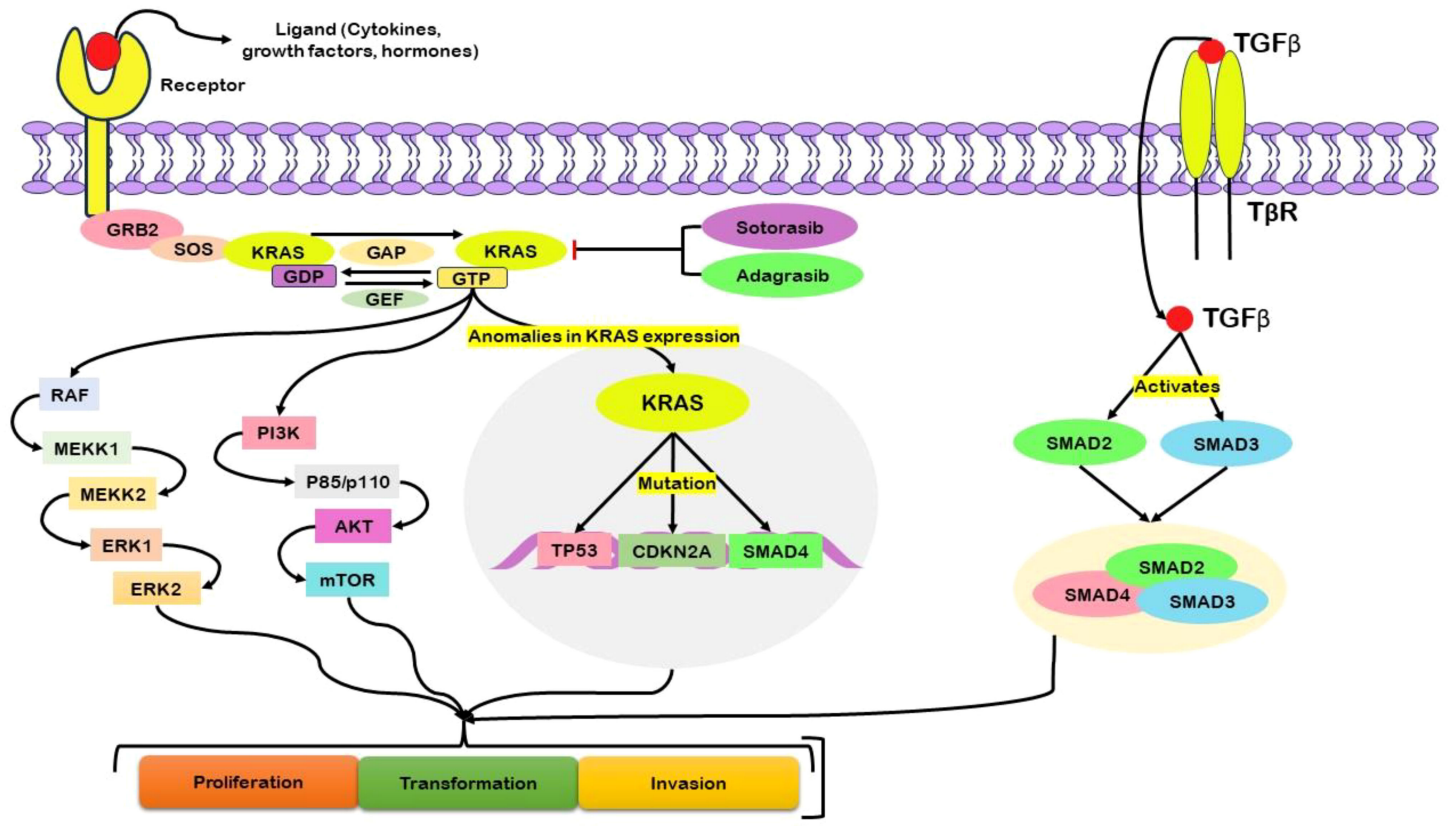
Schematic illustration of the central role of KRAS, particularly in its mutated form, in driving abnormal cellular activities associated with pancreatic cancer. Activation of growth factor receptors engages critical mediators, including growth factor receptor-bound protein 2 (GRB2), the guanine nucleotide exchange factor son of sevenless (SOS), and KRAS. Activation of KRAS, reliant on its membrane association and binding to guanosine triphosphate (GTP), initiates downstream signaling pathways. The complex regulation of KRAS GTP–guanosine diphosphate (GDP) cycling is governed by guanine nucleotide exchange factors (GEFs) and GTPase-activating proteins (GAPs). Mutations in KRAS disrupt this regulatory mechanism, resulting in persistent GTP binding and continuous downstream signaling. This dysregulation impacts nuclear transcription factors, influencing cellular proliferation, survival, and transformation. Abnormal expression of KRAS is associated with mutations in key genes such as TP53, CDKN2A, and SMAD4, further promoting oncogenesis. KRAS activity also relates to the activation of Transforming Growth Factor-beta (TGF-β) and subsequent downstream signaling. Therapeutic interventions targeting KRAS, such as Sotorasib and Adagrasib, are commonly used in the treatment of pancreatic cancer.

K-Ras plays a vital role in pancreatic ductal adenocarcinoma (PDAC), and K-Ras point mutations are highly prevalent among most PDAC patients. These mutations underscore fundamental genetic modifications originating in early pancreatic lesions, especially in low-grade PanIN (40). The persistent proliferation and survival of pancreatic cancer cells rely on the signaling activity of K-Ras (41). The initiation of the KRAS protein triggers its downstream intracellular pathways (**Figure 2**). Following the activation of growth factor receptors, such as tyrosine kinase or G-coupled receptors, growth factor receptor-bound protein 2 (GRB2) associates with the guanine nucleotide exchange factor son of sevenless (SOS) and engages with the KRAS protein (42). To be active, KRAS must be anchored to the cell membrane, where influential membrane association occurs. Once this association is established, KRAS becomes activated when it is bound to GTP (43).

Inherent KRAS GTP–GDP cycling is regulated by guanine nucleotide exchange factors (GEFs), which facilitate nucleotide exchange, and by GTPase-activating proteins (GAPs), which accelerate the intrinsic GTP hydrolysis activity of KRAS (44). In cases of KRAS mutation, the intrinsic GTPase activity is compromised, impeding the role of GAPs in facilitating the conversion of GTP to GDP (45). Consequently, KRAS remains persistently bound to GTP, initiating downstream signaling pathways. This, in turn, activates nuclear transcription factors, ultimately leading to cellular processes such as proliferation, survival, and transformation (46). The dysregulation of KRAS function due to mutation underscores its pivotal role in driving aberrant cellular activities associated with pancreatic cancer. Mutations in codons G12D or G12V cause acinar to ductal metaplasia and PanIN, which advances PDAC (47). Mutations in tumor suppressor genes, viz. P16/CDKN2A, SMAD4, and p53, combined with a positive K-Ras mutation, enhance cancer development in mouse models (48).

Various downstream effectors, including classical Raf/MAPK/extracellular signal-regulated kinase (Erk) (49), PI3Ks/(PDK-1)/Akt, RalGEFs, and phospholipase Cϵ, play crucial roles in the signaling cascade of K-Ras. Disruptions or mutations within these downstream cascades introduce complexities in K-RAS-driven PDAC (50). The presence of a persistently active oncogenic class 1A PI3K, such as PI3CA H1047R, hinders K-RasG12D-driven PDAC, triggers acinar to ductal metaplasia, and initiates precancerous PanIN while also precluding the involvement of PDK-1 (51). The most commonly used active suppressor in pancreatic cancer is P16/CDKN2A (52). It prevents retinoblastoma from being phosphorylated by CDK4/6, preventing cells from entering the S phase of the cell cycle (53).

Various factors contribute to P16/CDKN2A inhibition, such as epigenetic suppression and homozygous deletion, highlighting the critical role of this tumor suppressor gene in this disease (54). Moreover, the haploinsufficiency of P16/CDKN2A, especially in K-Ras-mutant mice, significantly advances the development of PanIN lesions and PDAC (55). SMAD4, another notable tumor suppressor gene, functions downstream of TGF-β signaling, exerting control over cell cycle progression and promoting apoptosis. TGF-β triggers the activation of Smad2 and Smad3, resulting in their binding with Smad4. Subsequently, this complex relocates to the nucleus, influencing gene expression (56).

In PDAC, SMAD4 loss promotes carcinogenesis and potentiates K-RasG12D-driven acinar to ductal metaplasia, PanIN, and PDAC (57). SMAD4 inactivation often occurs through homozygous deletion, highlighting its crucial role as a gatekeeper in pancreatic cancer (58). The tumor suppressor p53, encoded by TP53, is mutated in most pancreatic cancer patients (59). Interestingly, a heterozygous inactivating mutation (p53R172H/+) in combination with K-RasG12D amplifies PanIN and PDAC development in mouse models (60). Thus, p53 acts as a crucial barrier against K-Ras-driven pancreatic carcinogenesis. P53 regulates various cellular functions, including halting the cell cycle, facilitating DNA repair, inducing senescence, and promoting apoptosis (61). The deviant activation of K-Ras leads to mutations in TP53, CDKN2A, and SMAD4, which propels the development and progression of pancreatic cancer (62). These molecular mechanisms highlight the challenges in targeting K-Ras directly, as its mutational landscape and downstream signaling pathways are highly complex and context dependent. Drugs such as sotorasib (FDA-approved) and adagrasib target KRAS as therapeutic interventions for various types of cancer, including pancreatic cancer (63).

### Notch signaling

**Figure 3:**
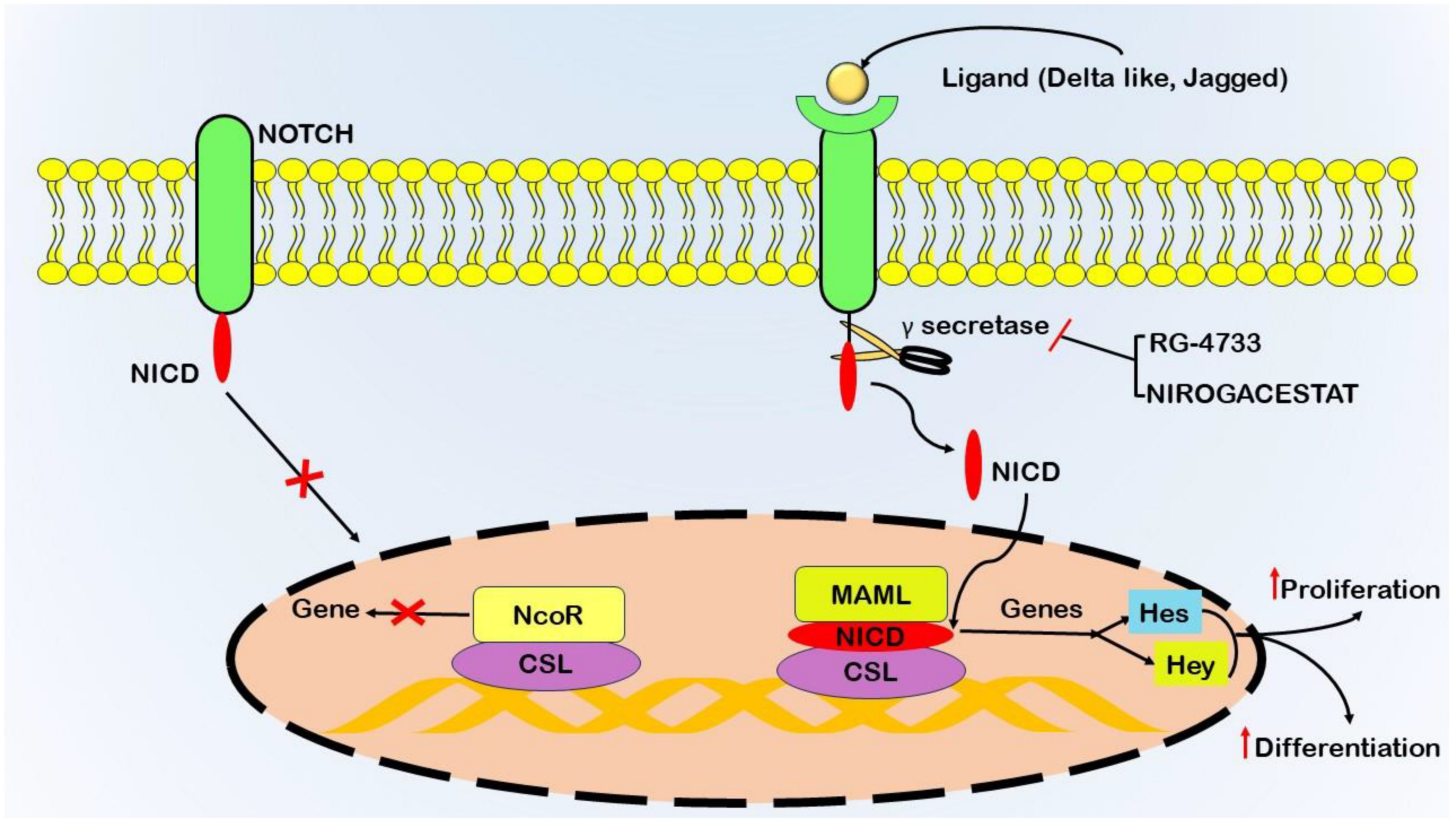
Schematic representation of gamma-secretase activity in initiating the Notch signaling pathway, a critical system involving transmembrane receptors (Notch1–4) and ligands (Delta-like 1, 3, 4, Jagged1, 2). Notch receptors on the cell surface interact with adjacent Delta and Jagged ligands, triggering sequential proteolytic cleavages. Tumor necrosis factor-alpha-converting enzyme (TACE) or ADAM10 mediates the initial cleavage, followed by the γ-secretase complex executing the second cleavage. This process releases the Notch intracellular domain (NICD) from the cell membrane, allowing its translocation to the nucleus. Inside the nucleus, NICD binds to the CSL transcription factor, displacing co-repressors and recruiting transcriptional activators such as Mastermind-like1 (Maml). This activation leads to the transcription of target genes Hes and Hey, which regulate cellular proliferation and differentiation. Gamma-secretase inhibitors (GSIs) impede the cleavage of Notch receptors by the γ-secretase complex, preventing NICD release and modulating Notch signaling. Therapeutic interventions, including drugs such as RG-4733 and Nirogacestat, act as inhibitors of γ-secretase.

The Notch signaling pathway significantly contributes to the pathogenesis of pancreatic cancer by precisely governing cellular processes, including proliferation, differentiation, and apoptosis, and plays a vital role in growth and tissue homeostasis (64). Dysregulation of Notch signaling promotes carcinoma initiation and onset (**Figure 3**). The human Notch family comprises five ligands (Delta-like 1, 3, 4, and Jagged 1, 2) and four receptors (Notch1-4) (65). The initiation of Notch signaling involves ligand-;receptor interactions, leading to the proteolytic cleavage of the Notch receptor by γ-secretase (66). During this stage, the Notch intracellular domain (NICD) is liberated, moves to the nucleus, and associates with Mastermind-like (MAML), CSL (CBF1/RBPJκ in mammals), and other coactivators. This associated assembly then stimulates the transcription of target genes, including those belonging to the Hes and Hey families (67).

Dysregulation of the Notch signaling pathway significantly contributes to tumorigenesis within the context of pancreatic cancer (68). Mutations that activate Notch receptors (NOTCH1 and NOTCH2) have been detected in a subset of pancreatic cancer cases, leading to ligand-independent activation of Notch signaling (69). Additionally, an increase in the expression of Notch ligands, namely, Jagged1 and Jagged2, further substantiates the dysregulation of this pathway in pancreatic cancer (70). The therapeutic target involves blocking the activity of γ-secretase. Drugs such as RG-4733 and nirogacestat are being tested in clinical trials as inhibitors of γ-secretase (71).

### Hedgehog signaling

**Figure 4:**
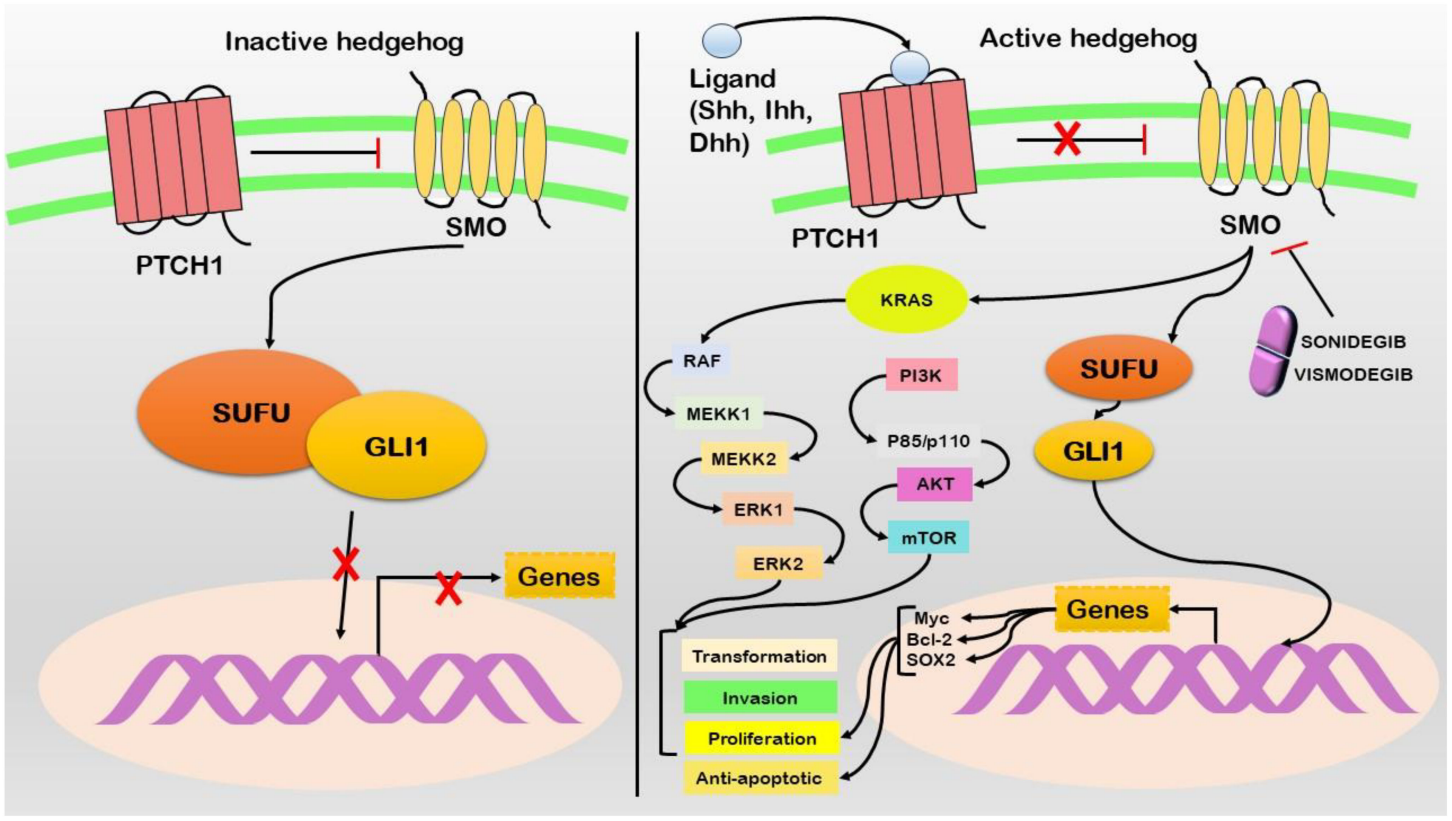
In the absence of Shh ligand (left), the pathway remains inactive with Patched1 (PTCH1) inhibiting Smoothened (SMO), resulting in the sequestration of GLI1 in the cytoplasm via Suppressor of Fused (SUFU). Upon the presence of Shh ligand (right), PTCH1 suppression of SMO is relieved, permitting GLI1 to accumulate in the nucleus. This activation induces the transcription of target genes, promoting various oncogenic properties. Active Hedgehog signaling leads to the activation of KRAS and its downstream signaling cascade. Therapeutic interventions such as Sonidegib and Vismodegib act as SMO inhibitors, thereby disrupting the pathway.

Hedgehog signaling has emerged as a pivotal pathway implicated in advancing pancreatic cancer. While this cascade is vital for embryogenesis and tissue homeostasis, it is associated with pancreatic cancer (72) (**Figure 4**). The Hedgehog pathway comprises three major components: Hedgehog ligands (Sonic Hedgehog SHH, Indian Hedgehog IHH, and Desert Hedgehog DHH) (73). Patched (PTCH) is a transmembrane receptor, while Smoothened (SMO) is a G protein-coupled receptor-like protein. PTCH inhibits SMO in the absence of Hedgehog ligands (74). However, when Hedgehog ligands bind to PTCH, SMO is released from inhibition, initiating downstream signaling events (75). The aberrant activation of Hedgehog signaling in pancreatic cancer is frequently linked to increased expression of the SHH ligand (76). Furthermore, SHH overexpression is observed in pancreatic cancer precursor lesions (PanIN) and invasive carcinoma (77). Genetic alterations in Hedgehog pathway components, including mutations in SMO and amplifications of GLI1 and GLI2 (downstream transcription factors), contribute to the expression of proproliferative and antiapoptotic genes such as *Myc*, *Bcl-2*, and *Sox2*. Importantly, Hedgehog signaling engages in crosstalk with other pathways, particularly K-Ras and Notch, thereby influencing the behavior of pancreatic cancer cells (78). This activation promotes cancer stem cell characteristics and significantly contributes to tumor initiation, progression, and therapeutic resistance development. Sonidegib and vismodegib function as inhibitors targeting the Smoothened (SMO) protein. These drugs work by interfering with the activity of SMO, a vital component of the Hedgehog signaling pathway (**Supplementary Table S1**).

### PI3K/AKT/mTOR signaling

PI3K/AKT/mTOR signaling governs cell survival, proliferation, and metabolism. Its dysregulation is common in pancreatic cancer, contributing significantly to its aggressive phenotype (79). PI3K triggers the activation of this pathway by phosphorylating phosphatidylinositol 4,5-bisphosphate (PIP2) and produces phosphatidylinositol 3,4,5-trisphosphate (PIP3) (80). PIP3 subsequently activates AKT, a serine/threonine kinase that phosphorylates various downstream targets, including the mammalian target of rapamycin (mTOR) (81). In pancreatic cancer, the PI3K/AKT/mTOR pathway frequently undergoes dysregulation due to genetic alterations. These alterations include mutations in PIK3CA, which encodes the catalytic subunit of PI3K; damage to the function of the tumor suppressor phosphatase and tensin homolog (PTEN); and activating mutations in AKT1 (82). These genetic changes result in sustained pathway activation, promoting cell survival, proliferation, and resistance to apoptosis. Furthermore, the PI3K/AKT/mTOR pathway engages in crosstalk with other signaling pathways, including K-Ras and Notch, further contributing to the overall complexity of pancreatic cancer signaling networks (83).

### Wnt/β-catenin pathway

The Wnt/β-catenin signaling pathway is a pivotal regulatory mechanism that governs diverse cellular processes, including cell proliferation, differentiation, and survival. Dysregulation of this pathway has been linked to the initiation and progression of various cancers, including pancreatic cancer (84). Pancreatic cancer exhibits complex molecular alterations, and disturbances in the Wnt/β-catenin signaling pathway significantly contribute to its pathogenesis. In the typical cellular environment of the pancreas, the destruction complex involves APC, Axin, GSK-3β, and CK1, coordinating the destruction of β-catenin (85). However, in pancreatic cancer, various mechanisms contribute to the abnormal initiation of the Wnt pathway. Wnt ligands, particularly Wnt2 and Wnt5a, are frequently overexpressed, initiating signaling through Frizzled receptors and LRP5/6 coreceptors. This binding event disrupts the destruction complex, hindering the phosphorylation and degradation of β-catenin (86). Stable β-catenin then translocates to the nucleus, where it forms a transcriptional complex with TCF/LEF transcription factors (87). This activation prompts the transcription of target genes, including MYC and Cyclin D1, which are pivotal for fostering uncontrolled cell proliferation and survival in pancreatic cancer (88). Genetic mutations further accentuate Wnt pathway dysregulation in pancreatic cancer. Mutations in APC or β-catenin result in constitutive activation of the pathway, emphasizing the genetic keystones of this aberrant signaling cascade (89).

The clinical significance of these molecular insights is highlighted by experimental approaches directing the Wnt/β-catenin pathway in pancreatic cancer (90). Investigations are underway on small molecule inhibitors that disrupt crucial components such as β-catenin or upstream regulators. Nevertheless, translating these promising preclinical discoveries into effective clinical interventions requires thorough examination through clinical trials that are tailored explicitly for patients with pancreatic cancer. Furthermore, research has shown the potential effectiveness of inhibiting the Wnt pathway in preclinical models of pancreatic cancer (91, 92). For instance, inhibiting Wnt signaling has been linked to reduced tumor growth and enhanced survival in murine models (93, 94). These observations offer a compelling rationale for exploring therapies targeting the Wnt pathway in the clinical context.

### Growth factor receptors

Pancreatic cancer is characterized by elevated levels of various mitogenic growth factors and their corresponding ligands. This includes heightened expression of epidermal growth factor (EGF) and its associated receptor, EGFR, multiple ligands that engage with EGFR, FGF and its receptor FGFR, insulin-like growth factor (IGF) and its receptor IGFR, platelet-derived growth factor (PDGF), and vascular endothelial growth factor (VEGF) (95). These signaling molecules are excessively expressed in pancreatic cancer, contributing to the aggressive nature of the disease.

#### Epidermal growth factor receptor in pancreatic cancer

In neoplastic cells, the activation of EGFR can occur inaccurately through various mechanisms, including ligand-dependent dimerization, point mutations, partial deletions, or overexpression (96). Increased expression of EGFR is linked to structural or numerical alterations of chromosome 7, where the EGFR gene is located (97). The c-ERBB-1 proto-oncogene encodes EGFR, and while in the normal pancreas, c-ERBB-1 is expressed exclusively in the islets of Langerhans, human pancreatic cancer cell lines frequently demonstrate its overexpression, which is observed in up to 85% of ductal adenocarcinomas (98). Pancreatic cancer is characterized by the accumulation of numerous genetic alterations, with early occurrences of KRAS mutations and EGFR gene amplification occurring during disease progression (99). Subsequent alterations involve *p16* inactivation, and late changes inactivate the *TP53* and *SMAD4* genes (100).

Importantly, ligands such as EGF and TGF-α play pivotal roles in EGFR activation. Following ligand binding, EGFR undergoes receptor homo or heterodimerization at the cell surface, followed by internalization. Dimerization leads to phosphorylation of the intracytoplasmic EGFR tyrosine kinase domain, which acts as a binding site for signaling molecules such as RAS (101). Activation of downstream pathways stimulates cellular proliferation, angiogenesis, and metastatic development and inhibits apoptosis (102). PDAC results from multiple mutations, with the initial precursor lesion being intraepithelial pancreatic neoplasia (PanIN). The progression from PanIN to invasive cancer involves sequential steps, starting from PanIN-1 with Kras mutation and telomere shortening to PanIN-2 with p16 inactivation and PanIN-3 with p53 and SMAD4 inactivation, culminating in invasive carcinoma (103). Acinar to ductal metaplasia (ADM) is considered a crucial precursor in PanIN progression (104).

Other noninvasive pancreatic neoplasms include mucinous cystic and intraductal mucinous neoplasms (105). Genome sequencing has identified four genes frequently implicated in PDAC: *Kras*, *CDKNA2A/p16*, *SMAD4*, and *TP53* (106). Kras oncogene mutations are predominant in PDAC, and their association with EGFR activation suggests a mechanism in which EGFR stimulation complements oncogenic pathways (107). Kras mutations hinder the ability of the Kras protein to hydrolyze guanosine triphosphate, maintaining the protein in an active signaling state that activates other pathways, such as the Raf and PI3 pathways (108).

#### Insulin-like growth factors and receptors

PDAC remains one of the most lethal cancer types due to its aggressive nature and resistance to conventional treatments. In the complex domain of pancreatic cancer progression, IGFs and their associated receptors have emerged as central regulators, influencing crucial processes such as angiogenesis, invasion, and cell survival (109). IGFs, particularly IGF-1 and IGF1R, significantly influence cancer biology. The IGF system is pivotal in regulating key processes essential for tumorigenesis and metastasis in various cancers, including pancreatic cancer (110). Increased expression of IGF-1 and IGF1R in PDAC is closely associated with unfavorable clinical outcomes, with elevated levels correlating with poor survival rates and higher tumor grades, establishing them as prognostic indicators for pancreatic cancer patients (111). *In vitro* investigations employing models of pancreatic cancer have provided valuable insights into the functional role of IGF-1 (112, 113).

Exogenous IGF-1 has been demonstrated to promote the development of pancreatic cancer cells, underscoring its function as a growth factor in disease progression (114). Furthermore, this growth-promoting effect can be counteracted by using antibodies designed to neutralize IGF-1, suggesting a potential avenue for therapeutic intervention. Despite promising preclinical findings, the translation of IGF1R-targeted therapies to clinical success has faced obstacles. Clinical trials, exemplified by the phase III trial investigating ganitumab, an antibody targeting IGF1R in conjunction with gemcitabine for metastatic pancreatic cancer patients, failed to yield a statistically significant improvement in survival (115). Amgen’s discontinuation of the trial underscores the challenges in translating preclinical success into meaningful clinical benefits (116). The setbacks in clinical trials targeting IGF1R in pancreatic cancer have raised critical questions about the complexities of the IGF system in the clinical context. Potential reasons for the lack of success may include adaptive resistance mechanisms, patient population heterogeneity or the influence of the tumor microenvironment. Future research endeavors should focus on identifying the roles of IGF signaling in pancreatic cancer, exploring combination therapies, and identifying potential biomarkers for patient stratification. While IGFs and their receptors drive the aggressive behavior of pancreatic cancer, the translation of knowledge into successful clinical interventions remains a formidable challenge in the field of medical research.

#### Fibroblast growth factor receptor signaling

FGFR signaling is pivotal for cellular processes, including proliferation, survival, and angiogenesis. Dysregulation of this pathway has been implicated in various cancers, including pancreatic cancer (117). A cascade of intracellular events occurs upon the binding of fibroblast growth factors (FGFs) to their corresponding FGFRs. This interaction initiates a structural alteration in the receptor, promoting the autophosphorylation of distinct tyrosine residues within the intracellular domain of FGFR. This autophosphorylation activates the receptor, creating docking sites for downstream signaling molecules (118). A crucial downstream target of activated FGFR is FGFR substrate 2 (FRS2). Upon FGF binding, FGFR initiates the phosphorylation of FRS2, a pivotal event in transducing signals to downstream pathways (119). Phosphorylated FRS2 is a scaffold for recruiting and activating components in two principal molecular pathways, the PI3K/Akt pathway and the rat sarcoma (Ras/MAPK) pathway, which are critical cellular signaling pathways (120). As a scaffold, phosphorylated FRS2 facilitates the recruitment and activation of key signaling molecules. In the PI3K/Akt pathway, activated FRS2 promotes the activation of PI3K, generating phosphatidylinositol (3,4,5)-trisphosphate (PIP3) and activating Akt, which is pivotal for cell survival and proliferation (121).

Phosphorylated FRS2 stimulates the Ras/MAPK pathway, which triggers the phosphorylation of mitogen-activated protein kinase kinase (MEK), triggering the activation of MAPK (ERK), which is renowned for its involvement in cellular proliferation and differentiation (122). Upregulation of the FGFR-1 and FGFR-2 receptors and increased expression of their ligands (FGF1-7) have been observed in a subset of pancreatic tumors. This dysregulation contributes to enhanced angiogenesis and mitogenesis, which are critical processes in cancer progression (123). The aberrant activation of FGFR signaling establishes an environment conducive to tumor growth and dissemination. Preclinical models of pancreatic cancer have demonstrated the therapeutic potential of inhibiting FGFR signaling. Approaches such as tyrosine kinase inhibitors, short hairpin RNA (shRNA) targeting FGFRs, and the administration of dovitinib have been explored. Inhibition of FGFR signaling in these models resulted in significant anticancer effects, suggesting that FGFR is a promising therapeutic target for pancreatic cancer (124–126).

#### Vascular endothelial growth factor

VEGF, a potent angiogenic factor, induces endothelial cell proliferation and sustains cell viability through engagement with its receptors, namely, VEGFR-1 and VEGFR-2 (127). In the context of PDAC, dysregulation of VEGF signaling contributes to establishing a proangiogenic microenvironment (128). Although PDAC is not traditionally highly vascularized, increased expression of VEGF mRNA has been consistently detected in tumor samples from PDAC patients. This upregulation correlates with disease progression and increased microvessel density, signifying an essential function for VEGF in fostering an angiogenic phenotype within the TME (129). These findings indicate that increased VEGF levels are associated with more aggressive tumor behavior, higher rates of metastasis, and poorer prognosis. The elevated microvessel density in response to heightened VEGF expression supports the notion that angiogenesis is a dynamic and critical process in PDAC progression (130).

Given the prominent role of VEGF in PDAC, therapeutic interventions targeting the VEGF pathway have garnered attention as potential strategies to prevent tumor growth and metastasis. In murine specimens, TNP-40, an analog of the antiangiogenic agent fumagillin, has demonstrated efficacy in reducing tumor growth and metastasis in PDAC cell lines (131). This preclinical evidence suggests that targeting angiogenesis through agents such as TNP-40 may have therapeutic implications for PDAC management. In preclinical studies involving pancreatic cancer, a viral vector containing PTK 787, a VEGFR tyrosine kinase inhibitor, has shown significant promise in impeding the metastasis and growth of PDAC (132). By specifically targeting the tyrosine kinase activity of VEGFR, PTK 787 interrupts downstream signaling cascades, mitigating the proangiogenic effects induced by VEGF (133). This approach holds the potential for developing targeted therapies that directly interfere with the VEGF–VEGFR axis, thereby impeding angiogenesis and disrupting the tumor’s ability to establish a robust blood supply. VEGF’s influence on endothelial cell proliferation and survival significantly contributes to the angiogenic microenvironment observed in PDAC. As research in this field progresses, the development of targeted therapies aimed at disrupting VEGF-mediated angiogenesis holds promise for improving outcomes in PDAC patients.

#### The receptor for advanced glycation end products in pancreatic tissue

The transmembrane receptor, receptor for advanced glycation end products (RAGE or AGER), is a member of the immunoglobulin superfamily and is located in the class III region of the major histocompatibility complex. Activation of this receptor has been linked to the initiation of inflammatory processes, which has implications for a spectrum of persistent ailments, such as hyperglycemia, brain degeneration disorders, and cancer (134). Recent studies have revealed the distinct roles of RAGE in pancreatic tumorigenesis and drug resistance, revealing novel therapeutic possibilities. Studies involving the suppression of RAGE expression, either through knockdown or knockout approaches, have demonstrated a notable delay in the growth of pancreatic tumors driven by oncogenic KRAS (135–137). This finding emphasizes that RAGE is a critical player in pancreatic cancer progression. In addition to its role in tumorigenesis, RAGE has emerged as a factor influencing drug resistance in pancreatic cancer (138). Suppression of RAGE has been associated with a reversal of drug resistance in experimental models (139), suggesting that RAGE, beyond its involvement in tumor initiation and growth, contributes to developing resistance mechanisms that often limit the effectiveness of therapeutic interventions in pancreatic cancer.

RAGE alters the interaction between antiapoptotic pathways, such as the IL6-pSTAT3 pathway, and autophagocytosis in the context of PDAC (140). Research involving the crossbreeding of conditional KRASG12D/+ mice prone to developing pancreatic cancer lesions with RAGE−/− knockout mice revealed a reduction in pancreatic lesions and prolonged survival compared to those of KRASG12D/+ RAGE+/+ mice (141). Another study revealed a progressive increase in RAGE protein levels as pancreatic lesions advanced, suggesting that RAGE is involved in PDAC initiation and disease progression (142). Additionally, heightened expression of RAGE was identified specifically within cancerous lesions, with no such elevation observed in neighboring normal tissue (143).

Two noteworthy RAGE ligands, namely, S100P and high mobility group box 1 (HMGB1), have undergone extensive examination in the context of pancreatic cancer (144). S100P, operating through a RAGE-dependent mechanism, stimulates the proliferation and migration of human pancreatic cancer Panc-1 cells (145). Moreover, S100P has been shown to exhibit protective effects against the cytotoxicity of 5-fluorouracil in Panc-1 cells (146). Additionally, RAGE activation by HMGB1 was linked to enhanced tumor growth, promoting the persistence of cancer cells by upregulating autophagocytosis and inhibiting apoptosis (147).

## Epithelial-mesenchymal transition

A substantial proportion of pancreatic cancer-related deaths can be attributed to the pivotal role played by EMT in the rapid progression of metastatic disease (148). Throughout EMT progression, epithelial cells undergo a profound transformation characterized by the loss of epithelial markers such as E-cadherin, occludin, claudin, and laminin-1 while concurrently gaining mesenchymal markers such as N-cadherin, vimentin, and fibronectin (149). This phenotypic shift is a hallmark of EMT and is linked to cancer cell invasion and metastatic potential. Dynamic alterations in cellular identity are essential for the metastatic cascade because they allow cancer cells to detach from the primary tumor, infiltrate surrounding tissues, enter the bloodstream, and colonize distant organs, particularly the liver (150). There are three distinct types of EMT, and their occurrence is context-dependent. Type 3 EMT, which is observable in carcinoma cells, is relevant for invasion and metastasis during tumor development (151). The activation of EMT mechanisms in carcinoma cells underscores its pivotal role in promoting the aggressive and metastatic behavior observed in pancreatic cancer. Hyaluronic acid and collagen are examples of insoluble components (152). Soluble elements in the extracellular matrix, including Wnt, FGF, HGF, Notch, TGF-β family members, TNF-α, and HIF1-α, synergistically contribute to cancer progression by guiding the EMT process. These components create a dynamic microenvironment that helps epithelial cells transdifferentiate into mesenchymal phenotypes (153). Crucial signaling pathways regulating EMT involve activating transcription factors such as Zeb-1 and 2, Snail 1 and 2, and members of the bHLH family (E12, E-47, and Twist). These transcription factors play a central role in composing the molecular changes associated with EMT (154). Furthermore, repression of the E-cadherin encoder (CDH1 gene) has emerged as a shared feature among these transcription factors (155).

TGF-β is a crucial mediator of EMT in a variety of tumors. The conventional TGF-β signaling pathway involves the binding of TGF-β to a type II receptor, which enables the transactivation of type I receptor (TβR I) (156). The serine/threonine kinase TβR I phosphorylates SMAD2, resulting in the association of SMAD2 with SMAD4. After nuclear translocation, this complex regulates target gene transcription (157). The activation of the transcription factors Snail, Zeb-1, Slug, and Twist is pivotal for the TGF-β-mediated induction of EMT (158). In PDAC, TGF-β may engage a noncanonical pathway, including the PI3K, ERK/MAPK, p38, RhoA, JNK, and other signaling pathways (159). EMT responses in the Colo357 pancreatic cancer cell line were not affected by RNA interference-induced SMAD4 knockdown (160). However, in alternative pancreatic cancer cell lines, the induction of TGF-β-mediated EMT was efficiently suppressed by the MEK-1 inhibitor PD98059 (161).

When a Wnt ligand is not present, β-catenin sequestration is regulated through a degradation component comprising Axin, adenomatous polyposis coli, glycogen synthase kinase-3 (GSK-3), and CK-1 (162). This process begins with CK-1 phosphorylating β-catenin at Ser45 (163). GSK-3 activates β-catenin by phosphorylating it at Thr41, Ser33, and Ser37. This phosphorylation event triggers ubiquitination, and subsequently, β-Trcp facilitates the proteasomal degradation of β-catenin (164). The systematic elimination of β-catenin prevents its nuclear buildup, impeding interaction with DNA-bound TCF/LEF complexes and histone deacetylase (HDAC) activity, ultimately suppressing Wnt target genes (165). Wnt ligands bind to the Frizzled and LRP5/6 receptors, causing a complex to develop, phosphorylating LRP5/6, stabilizing Axin, and facilitating GSK-3 complex disassembly. This process inactivates cytosolic β-catenin, allowing it to form a complex with TCF/LEF in the nucleus, thereby regulating genes crucial for cell growth and proliferation (166).

In addition to its role in β-catenin regulation, GSK-3β also promotes the phosphorylation and proteasomal degradation of Snail (167). Conversely, Wnt suppresses GSK-3β activity, causing increased Snail protein levels (168). K-Ras-induced activation of the Wnt/β-catenin pathway upregulates EMT stimulators in cancer cells (169). By decreasing the expression of Slug and Twist, reinstatement of Wnt inhibitory factor 1 causes a reduction in the levels of mesenchymal markers and an increase in epithelial indicators. Inhibition of β-catenin through the use of small hairpin RNA results in increased expression of E-cadherin, coupled with a decrease in the levels of mesenchymal markers such as vimentin, N-cadherin, and MMP-2 (170).

The Notch signaling pathway, which is integral to tissue development and apoptosis, encompasses four Notch receptors and five Notch ligands (Delta-like 1, 3, 4, Jagged-1, and 2) (171). Activation ensues upon the binding of the Notch protein to a neighboring cell’s receptor, initiating proteolytic cleavage facilitated by metalloproteases, TNF-α converting enzymes, and γ-secretase (66). The resulting active Notch intracellular domain fragment (NICD) translocates to the nucleus, where it forms a CSL-NICD complex with the transcription factor CSL (CBF1, a suppressor of Hairless, and Lag-1) (172). Functioning as a coactivator, this complex recruits additional coactivators, including p300, activating Notch target genes pivotal in governing cellular processes such as growth, proliferation, angiogenesis, and programmed cell death (173). Noteworthy target genes implicated in solid and hematological cancers include Cyclin D1, COX-2, Akt, MMP9, ERK, VEGF, c-Myc, mTOR, NF-κB, p53, p27, and p21 (174, 175). The Notch signaling pathway directly induces EMT by activating Slug and Snail-1 (176). The depletion of Notch-2 or midkine suppresses EMT in pancreatic cancer cells through Notch-2-mediated mechanisms (177).

### Growth factors and EMT

**Figure 5:**
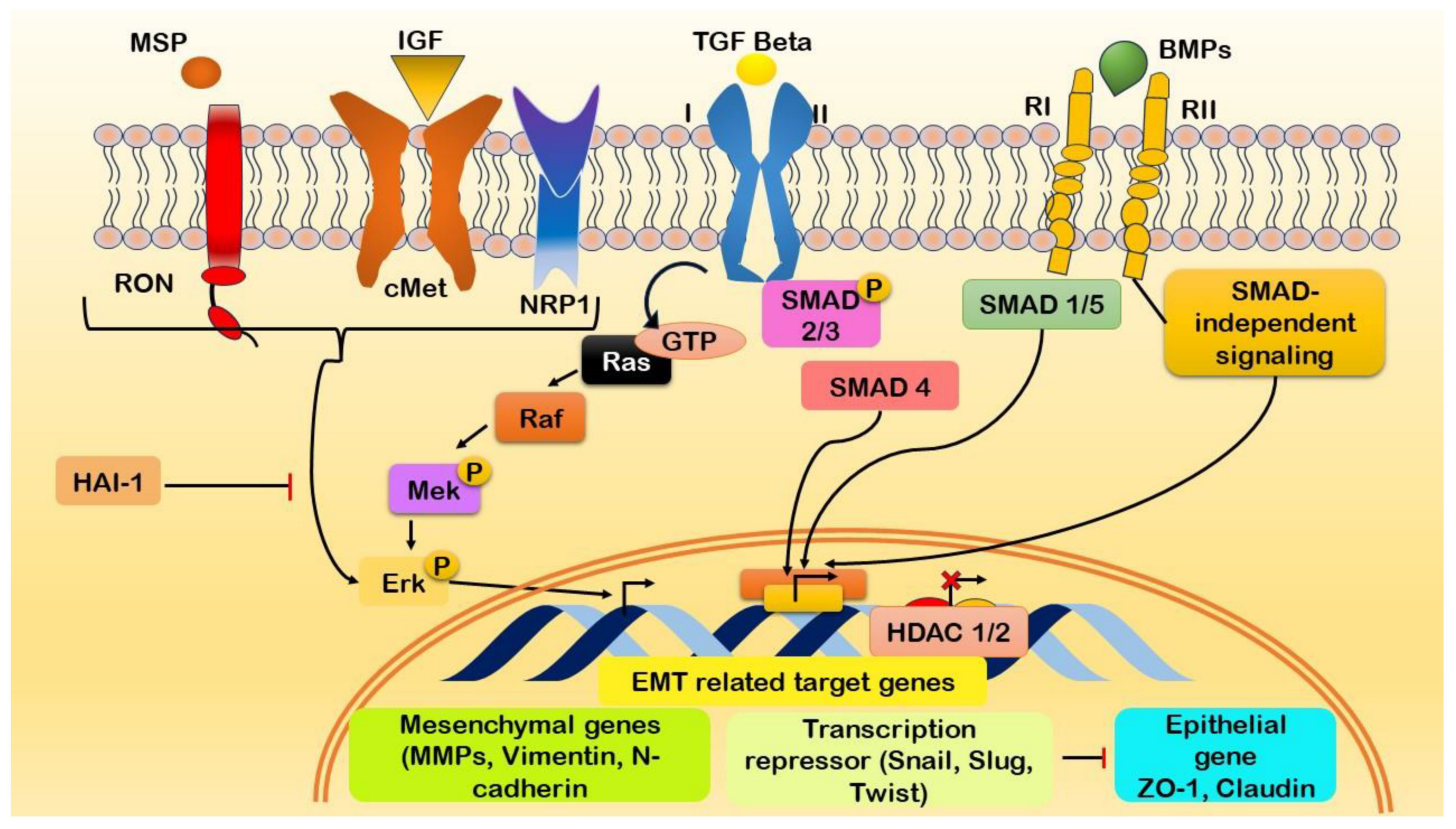
Illustration of the initiation of epithelial-mesenchymal transition (EMT) in pancreatic cancer through the activation of growth factor signaling cascades and the modulation of EMT-associated genes. Interaction between growth factors and their respective receptors initiates the expression of genes associated with EMT. Key molecular players involved in this process include Macrophage-Stimulating Protein (MSP), Insulin-like Growth Factor 1 (IGF1), Transforming Growth Factor-beta (TGFβ), Bone Morphogenetic Proteins (BMPs), Recepteur d’Origine Nantais (RON), Neuropilin-1 (NRP1), Ras-like GTPases (Ra1), Extracellular signal-Regulated Kinases (Erk), Histone Deacetylases 1/2 (HDAC1/2), Msh Homeobox 2 (MSX2), S100 Calcium-Binding Protein A4 (S100A4), and Zonula Occludens-1 (ZO-1). The complex interaction among these molecular components promotes the induction of EMT in pancreatic cancer.

The initiation of EMT in pancreatic cancer involves relationships among distinct molecular entities, each contributing unique functions to the dynamic process (**Figure 5**). Major surface protease (MSP) collaborates with IGF1 to induce cellular growth and survival, while tumor growth factor β (TGFβ) orchestrates alterations in cell morphology and promotes invasiveness (178). This process is complemented by bone morphogenetic proteins (BMPs), which influence cell differentiation and apoptosis, thus impacting the plasticity of cancer cells undergoing EMT (179). Recepteur d’Origine Nantais (RON) plays a pivotal role in influencing cell motility and invasion by interacting with neuropilin 1 (NRP1), which, in turn, contributes to angiogenesis and neural guidance within the TME (180). Retinoic acid-induced 1 (Ra1) influences cell cycle progression in concert with extracellular signal-regulated kinase (Erk), a key player in signaling cascades that transduce external signals to the nucleus, thereby affecting the cellular changes observed during EMT (181). Histone deacetylases 1 and 2 (HDAC1/2) modulate gene expression through epigenetic regulation, while Msh homeobox 2 (MSX2) influences cell differentiation and migration (182). S100 calcium binding protein A4 (S100A4) impacts cytoskeletal dynamics and motility and is crucial for the migratory aspects of EMT (183). ZO-1 contributes to cell adhesion and polarity through its role in tight junctions (184).

### Hippo signaling pathway

Pancreatic cancer, a formidable challenge in oncology, demands a comprehensive understanding of the underlying molecular pathways. The Hippo signaling network is a conserved system that governs cellular proliferation, organ growth, and regenerative processes (**Table 1**). At its core are the serine/threonine kinases MST1, MST2, LATS1, and LATS2 (200). MST1 and MST2 phosphorylate and activate the LATS1 and LATS2 kinases in collaboration with SAV1 (201). Subsequently, MOB1 binds to LATS1 and LATS2, leading to the phosphorylation of the Hippo transducers YAP and TAZ (202). This phosphorylation impedes the accumulation of YAP and TAZ in the nucleus and their interaction with TEAD transcription factors (201). When the regulatory module is inactive or when independent stimuli activate YAP/TAZ, these molecules translocate to the nucleus, where they engage with transcription factors (203). This interaction initiates the transcription of target genes, including CTGF, CYR61, ANKRD1, BIRC5, and AXL (204). Mechanical stimuli in the cellular environment (mechano-transduction), soluble substances, and metabolic pathways collectively impact the Hippo signaling pathway. Additionally, the system extensively communicates with other signaling pathways, such as the TGF-beta, Wnt, Sonic Hedgehog, and Notch pathways (205).

**Table 1:** The Hippo signaling pathway regulates cell processes involving MST1, MST2, LATS1, and LATS2 kinases.

Hippo Signaling Component	Description	Function	References
MST1 and MST2 (HPO in Drosophila)	Serine/threonine kinases constitute the foundational mechanisms of the Hippo cascade and collaborate with Salvador homolog 1 (SAV1) to execute the phosphorylation and activation of LATS1 and LATS2 kinases.	Initiates the phosphorylation cascade that regulates the downstream elements of the Hippo transduction. Prevents nuclear accumulation and communication of YAP and TAZ with transcription factors.	(185)
LATS1 and LATS2	Large tumor suppressor 1 and 2 kinases activated by MST1 and MST2. Combine with MOB kinase activator 1 (MOB1) to phosphorylate Hippo transducers YAP and TAZ.	The phosphorylation of YAP and TAZ obstructs their accumulation in the nucleus and their engagement with transcription factors, limiting their ability to carry out transcriptional activities.	(186)
SAV1	Salvador homolog 1 works with MST1 and MST2 to activate LATS1 and LATS2 kinases.	Facilitates the phosphorylation and stimulation of LATS1 and LATS2 kinases	(187)
MOB1	Adaptor protein that interacts with LATS1 and LATS2 kinases.	Forms a complex with LATS1 and LATS2, leading to the phosphorylation of YAP and TAZ in the Hippo signaling pathway.	(188)
YAP	Hippo transducer, upon phosphorylation, causes inhibition in the nuclear buildup and communication with transcription factors, including TEAD1, TEAD2, TEAD3, and TEAD4.	Phosphorylation by LATS1 and LATS2 inhibits YAP’s transcriptional activity.	(189)
TAZ (Transcriptional co-activator with PDZ-binding motif)	The Hippo transducer, akin to YAP, undergoes regulation through phosphorylation. Phosphorylated TAZ experiences inhibition in nuclear accumulation and interaction with transcription factors.	Phosphorylation by LATS1 and LATS2 inhibits TAZ’s transcriptional activity.	(190)
TEAD (TEA Transcriptional Factor)	Transcription factors that interact with YAP and TAZ when not phosphorylated.	YAP and TAZ, in their non-phosphorylated state, interact with TEAD transcription factors, directing the transcription of target genes associated with cell proliferation and growth.	(191)
Connective Tissue Growth Factor (CTGF)	Target genes of the Hippo pathway under the regulation of YAP and TAZ.	Expression of CTGF is mediated by YAP and TAZ when not phosphorylated, contributing to cell proliferation and tissue growth.	(192)
Cysteine-rich Angiogenic Inducer 61 (CYR61)	YAP and TAZ regulate the target gene of the Hippo pathway.	CYR61 expression is influenced by YAP and TAZ, playing a role in angiogenesis.	(193)
Ankyrin Repeat Domain 1 (ANKRD1)	The Hippo pathway, subject to regulation by YAP and TAZ, influences target genes.	ANKRD1 expression is modulated by YAP and TAZ, contributing to various cellular processes.	(194, 195)
Baculoviral Inhibitor of Apoptosis Repeat-containing 5 (BIRC5)	YAP and TAZ regulate the target gene of the Hippo pathway.	BIRC5 expression is influenced by YAP and TAZ, playing a role in apoptosis regulation.	(196)
AXL Receptor Tyrosine Kinase (AXL)	YAP and TAZ regulate the target gene of the Hippo pathway.	AXL expression is modulated by YAP and TAZ, influencing cellular responses.	(197)
Inputs Control Hippo Signaling	A variety of inputs, including mechanical cues from the cellular surroundings, soluble substances, and pathways related to metabolism.	Multiple external factors, such as mechanical signals, soluble factors, and metabolic pathways, influence Hippo signaling. These inputs play a role in controlling cellular proliferation and development.	(198)
Crosstalk with Other Signaling	The Hippo pathway interacts with different signaling pathways, including transforming growth factor-beta, Wnt, Sonic Hedgehog, and Notch.	Interactions with multiple signaling pathways coordinate cellular processes, encompassing cell proliferation and differentiation.	(199)

It controls YAP and TAZ translocation, influencing target gene transcription and crosstalk with various signaling pathways.

### Snail transcription factors

Snail-1 and Snail-2 are transcription factors that play pivotal roles in regulating the initiation of EMT, a crucial process implicated in the progression and metastasis of pancreatic cancer. These transcription factors are characterized by their conserved C2H2-type zinc finger motifs and the essential Snail1/GFI domain at the amino terminus, which is critical for maintaining the transcriptional suppression of target genes and protein stability (206, 207). In PDAC, Snail and its closely related family member Slug have emerged as key mediators of EMT. Furthermore, Slug is present in 50% of PDAC patients, while Snail expression is detected in a striking 68% of cases (208). Elevated Snail expression levels in pancreatic cancer have been associated with lymph node invasion and distant metastasis, underscoring its role in promoting invasive and metastatic behavior. When pancreatic cancer cell lines are transfected with Snail, they exhibit increased invasive and metastatic potential in orthotopic pancreatic cancer models, manifesting EMT characteristics during the invasive phase of tumor progression (209).

Importantly, the inhibition of Snail amplifies the response to the chemotherapeutic agent gemcitabine and contributes to extended overall survival in a murine model engineered for PDAC (210). This finding highlights the potential therapeutic benefits of targeting Snail in pancreatic cancer treatment. The mechanisms by which Snail exerts its pro-metastatic effects in pancreatic cancer involve suppressing genes crucial for maintaining the epithelial phenotype, such as occludin, E-cadherin, claudin, and cytokeratin-18, while simultaneously promoting the expression of mesenchymal genes like N-cadherin, vimentin, and fibronectin (211). Moreover, Snail governs the expression of genes linked to apoptosis (P53, BID, and DFF40) and cell polarity (Crumbs3, Lgl2, and dlg3), with a particular emphasis on downregulating the key epithelial marker E-cadherin (212).

### Zeb transcription factors

Numerous studies have examined the Zeb family of transcription factors, demonstrating their important function as strong EMT inducers (213). Interestingly, there is a positive correlation between elevated Zeb-1 expression in the tumor-associated stroma and pancreatic cancer cells and a poor prognosis for individuals with PDAC. Examination of human tissue specimens and pancreatic cancer cell lines revealed a connection between Zeb-1 and the expression of E-cadherin (214). Inhibition of Zeb-1 has been associated with notable decreases in cell migration, tumorigenesis, and dissemination (215). Research indicates that decreased expression of essential components related to epithelial development, cellular adhesion, and cellular polarity is a recognized consequence of heightened Zeb-1 expression (216). Specifically, Zeb-1 selectively engages either HDAC-1/2 or the switch/sucrose nonfermentable chromatin remodeling protein BRG1 at the promoter region of the CDH-1 gene, resulting in a reduction in E-cadherin synthesis (217). Consequently, inhibiting Zeb-1 has emerged as a potentially impactful treatment strategy for individuals with PDAC.

### bHLH Transcription factors

bHLH proteins, including E12, E47, Twist 1, and Twist 2, which are essential EMT players (218), have been investigated. EMT is actively promoted by E47 and E12, which suppress the production of E-cadherin (219). Twists 1 and 2, which have been identified as the primary regulators of EMT during pathogenesis, play important roles (220). Patients with PDAC typically have very weak or no Twist expression in their samples (221). Comparably, whereas Twist expression is enhanced under hypoxic conditions, pancreatic cancer cell lines such as PANC-1, MiaPaCa-2, Capan-1, AsPC-1, and HPAF-2 cells exhibit low Twist expression, suggesting a possible role for Twist in the invasive nature of pancreatic tumors (222). Twist has been linked to decreased E-cadherin expression and increased N-cadherin expression (223). Twist engages with various elements of the Mi2/nucleosome remodeling and deacetylase complex, contributing to the inhibition of E-cadherin transcription (224).

## The tumor microenvironment in pancreatic cancer

TME is characterized by distinct physical and biochemical properties that promote interactions between stromal and malignant cells to drive metastasis, carcinogenesis, disease progression, and resistance to treatment (225). In addition to the resistance linked to desmoplasia, pancreatic cancer is characterized by a very immunosuppressive environment with several components and processes that obstruct efficient immune responses directed against malignancy (226). Due to the many immunological regulatory cells that enter the pancreatic cancer stroma, the principal processes of the TME are challenging to understand. Important TME constituents include soluble factors, immune cells, acellular stroma, and pancreatic stellate cells (227). Desmoplasia, a condition in which hyperactive cancer-associated fibroblasts deposit abnormal ECM, primarily fibrillar type I collagen, is a characteristic of PDAC (228). Disruption of cell-ECM homeostasis and stromal remodeling are linked to treatment resistance and metastasis during cancer progression (229). A thorough mechanistic understanding of PDAC pathophysiology requires additional sophisticated *in vitro* and *in vivo* models owing to the critical interactions between the tumor and the stromal extracellular matrix. The roles of the TME, constituents, and consequences in PDAC are listed in **Table 2**.

**Table 2:** Tumor microenvironment characterization, constituents, and consequences in pancreatic ductal adenocarcinoma.

TME Component	Description	Role in Pancreatic Cancer	References
Immune Cells	Various immune regulatory cells in the heterogeneous pancreatic cancer stroma contribute to an immunosuppressive environment.	Contribute to therapeutic resistance and influence immune responses.	(230)
Pancreatic Stellate Cells	Cancer-associated fibroblasts in a heightened state deposit a substantial extracellular matrix (ECM), predominantly consisting of fibrous type I collagen, leading to the development of desmoplasia.	Involved in the advancement of cancer, the spread of cancer cells, and resistance to drugs.	(231)
Acellular Stroma	The extracellular matrix (ECM) lacks cellular components, a key element of desmoplasia in pancreatic cancer.	Contributes to the remodeling of the stroma and the dysregulation of cell-ECM homeostasis.	(232)
Soluble Factors	Various signaling molecules and cytokines in the TME influence cell behavior and communication between oncogenic and stromal cells.	Contribute to the advancement of the disease and resistance to therapeutic interventions.	(233)
Basement Membrane (BM)	A slender, sheet-like arrangement primarily composed of laminin, non-fibrous type IV collagen, and heparan sulfate proteoglycan acts as a protective and polarizing barrier for layers of epithelial cells.	Important in maintaining epithelial cell integrity and polarization	(234)
Interstitial Matrix (IM)	The ECM, rich in fibrillar type I collagen, accommodates distinct mesenchymal cells like fibroblasts.	Crucial for mesenchymal cell support and function	(235)
Collagen Density	Collagen density, a significant component of the ECM, influences tumor-infiltrating T-cell activity and may regulate immune evasion by cancer cells.	Modulates T cell activity, affecting the expression of cytotoxic and regulatory markers	(236)
Fibronectin	Protein bridges collagens and integrins, promoting collagen activity and implicated in the infiltration of PDAC cells into the basement membrane.	Contributes to the spite of PDAC cells and the process of fibrogenesis.	(237)
Hyaluronan (HA)	Richly gathered in the stroma of malignant tumors, including PDAC, associated with tumor progression, promoting various cancer-related processes.	Linked to cellular activities such as proliferation, migration, invasion, metastasis, blood vessel formation, and resilience against chemotherapy.	(238)
Tumor-Infiltrating Lymphocytes (TILs)	CD8+ T lymphocytes and CD4+ helper T lymphocytes, where CD8+ is linked with favorable outcomes, while CD4+ helper T2 lymphocytes negatively impact patient survival.	Play a crucial role in immune responses, influence patient prognosis	(239)

The interstitial matrix (IM) and basement membrane (BM) make up the ECM found in both PDAC and normal tissues (240). BM is a thin, sheet-like structure that provides polarization and protection to epithelial cell layers. It primarily comprises laminin, nonfibrillar type IV collagen, and heparan sulfate proteoglycans (241). In contrast, specific mesenchymal cells, such as fibroblasts, inhabit the IM and are primarily composed of fibrillar type I collagen (242). According to one study, collagen density may play a role in cancer cells’ ability to evade the immune system by acting as a unique anticancer T-cell function controller in three-dimensional T-cell culture (243). The expression of cytotoxic and regulatory markers is influenced by collagen density, which also affects the activity of T lymphocytes that infiltrate tumors (243).

Like collagens, fibronectin has distinct impacts on the biology of prostate cancer and serves as a connecting protein between integrins and collagens, promoting the function of collagens (244). Fibronectin promotes the malignancy and fibrogenesis of PDAC cells, as evidenced by its involvement in pancreatic stellate cell ECM creation and PDAC cell penetration into the basement membrane (245).

In the stroma of malignant tumors, including PDAC, hyaluronan (HA), a significant ECM component, accumulates abundantly. This accumulation is associated with the advancement of tumors, as it stimulates cellular proliferation, movement, infiltration, metastasis, angiogenesis, and resilience to chemotherapy (246). According to research, HA and its receptors are overexpressed in PDAC, and abnormal HA buildup is associated with a poor prognosis (247). Therefore, targeting HA may have therapeutic benefits in the treatment of PDAC. The TME is maintained by ongoing interactions among cells and between cells and the extracellular matrix, and the initiation of interactions between epithelial cells, pancreatic cancer cells, and stromal cells in the TME is critical for drug resistance and the progression of connective tissue in primary and metastatic locations (248).

TME components can promote EMT and angiogenesis, which contribute to the capacity of pancreatic cancer to spread. Furthermore, the TME complicates immunotherapeutic treatments (249). Tumor-infiltrating lymphocytes (TILs), including CD8+ T cells and CD4+ helper T1 lymphocytes, are related to positive outcomes, whereas CD4+ helper T2 lymphocytes are linked to unfavorable patient survival (250). Immune and inflammatory cells play essential roles in the TME of pancreatic cancer, contributing to chemotherapy resilience and serving as early contributors to carcinogenesis and metastasis.

## Therapeutic approaches for pancreatic cancer

Pancreatic cancer poses a significant challenge in oncology because of its aggressive behavior and restricted treatment modalities. This study examined an integrative strategy that combines conventional medical interventions with complementary and alternative therapies to improve the comprehensive well-being of individuals with pancreatic cancer.

### Noncoding RNA

Noncoding RNAs (ncRNAs) are a different family of molecules that play critical regulatory roles in several life processes, including pathological illnesses such as cancer, cardiovascular disease, and neurodegenerative disorders (251). MicroRNAs (miRNAs) and synthetic antagomirs, which have an approximate length of 22 nucleotides, are crucial in the delicate arrangement of cellular processes and significantly influence cellular proliferation, apoptosis, and autophagy (252). Among them is miR-203, which has received attention for its suspected anticancer effects via precise gene expression control (253). Furthermore, the discovery of circulating miRNAs with possible biomarker value offers promise for noninvasive surveillance of the dynamic evolution and severity of pancreatic cancer (254). MiRNAs such as miR17-92 (255) and miR-21 limit cellular growth (256), while miR-126 acts as an antioncogene (257). Additional complexities emerge with miR-15b and miR-155, which are involved in mutation accumulation (258), and with miR-10b and miR-29, which are critical for triggering metastatic pathways (259). The complex interaction includes miRNAs such as let-7d, miR-23b, miR-126, and miR-200c, which promote inflammatory responses (260, 261), and miR-21 and miR17-92, which decrease immune cell clearance (262). Let-7, miR-16, miR-21, and miR-221/222 all play roles in the maintenance of replicative immortality, demonstrating the extensive regulatory networks mediated by these small RNA species (263–266).

MiR-203 has emerged as a crucial regulator in pancreatic cancer, limiting cell invasion and migration through the targeted control of caveolin-1 (267). Its downregulation in pancreatic cancers emphasizes its importance in disease genesis. Other miRNAs, such as miR-21, miR-155, miR-221, miR-222, miR-376a, and miR-301, contribute significantly to tumorigenic qualities by altering the expression of DJ-1 and affecting the PTEN-PI3K/AKT pathway (268). In addition to this complication, miR-203 has dual functions in pancreatic cancer, limiting cell proliferation while simultaneously promoting apoptosis via precise changes in the expression of suppressor of cytokine signaling 3 (SOCS3) (269). However, the specific molecular processes and crucial functions of miR-203 in pancreatic cancer remain unknown.

### Chemotherapy

Pancreatic cancer therapy presents a daunting challenge, as a multimodal strategy that considers the disease stage, the patient’s general health, and the development of research is needed. Surgical intervention is often the first option for resectable tumors, with the possibility of a cure if the cancer is restricted to the pancreas. Adjuvant chemotherapy becomes critical after surgery, with regular use of medicines such as gemcitabine, fluorouracil (5-FU), capecitabine, oxaliplatin, and irinotecan (270). Oxaliplatin, a platinum-based drug, induces cross-linking in DNA, affecting the nucleotide excision repair (NER) cascade and activating the DNA damage response (DDR) pathway (271). Erlotinib, an oral EGFR inhibitor, disrupts essential signaling pathways and is particularly effective in treating tumors with EGFR abnormalities (272).

Gemcitabine targets the deoxycytidine pathway and affects the nucleotide pool, mainly affecting the cell cycle and DNA synthesis. The DNA synthesis and repair route is the primary signaling mechanism affected by gemcitabine (273). Gemcitabine is a nucleoside analog that has structural similarities with DNA. During replication, gemcitabine enters the cell, becomes phosphorylated, and joins the growing DNA chain. This insertion stops the DNA chain from elongating and stops further synthesis from occurring. Gemcitabine thus causes cell cycle arrest in the S phase, the stage at which DNA synthesis occurs (274).

A series of events, such as activating cell cycle checkpoints and DNA damage response pathways, are initiated when DNA replication stalls. Additionally, gemcitabine prevents the manufacture of deoxyribonucleotides, which are necessary building blocks for DNA replication, by inhibiting ribonucleotide reductase (275). Gemcitabine further inhibits DNA synthesis by reducing the intracellular pool of deoxyribonucleotides (276). By targeting the dynamics of microtubules inside cells, nab-paclitaxel affects signaling pathways linked with microtubules (277). The ability of paclitaxel, the active ingredient of nab-paclitaxel, to stabilize microtubules is its primary mode of action. Dynamic structural elements of cytoskeleton microtubules are essential for many cellular functions, including mitosis (278).

In particular, nab-paclitaxel disrupts the normal dynamics and function of microtubules by interfering with their disintegration during mitosis. This perturbation stops the cell cycle in the G2/M phase, triggering apoptosis or programmed cell death (279). Nab-paclitaxel disrupts the mitotic spindle machinery necessary for appropriate chromosomal segregation during cell division by targeting microtubules and altering their regular movements (280). Although nab-paclitaxel primarily affects microtubule stability and the accompanying effects on cell cycle progression, it also indirectly affects several signaling pathways linked to cell survival and division (281). Targeting the thymidylate synthase enzymes 5-fluorouracil (5-FU) and capecitabine—essential drugs for treating different types of cancer—has a similar mechanism of action that involves interfering with DNA synthesis (282). When administered intravenously, 5-FU acts as an antimetabolite, inhibiting DNA replication and repair by impeding the transformation of deoxyuridine monophosphate (dUMP) to deoxythymidine monophosphate (dTMP) (283).

The main protein target of 5-FU is thymidylate synthase. When this enzyme is inhibited, many biological reactions are triggered, including the activation of cell cycle checkpoints, DNA damage response pathways, and death (284). In contrast, capecitabine is an oral prodrug that enters tumor cells and proceeds via enzymatic conversions to produce 5-FU (285). Like 5-FU, which is delivered directly, 5-FU inhibits thymidylate synthase once it is converted to exert its antimetabolite effects (286). The protein target for thymidylate synthase remains constant, causing errors in DNA synthesis and other cellular reactions that result in cell cycle arrest and death (287). Oxaliplatin is a platinum-based chemotherapeutic drug that mainly targets guanine nucleotides in genomic DNA by forming covalent DNA adducts (288). DNA strands become cross-linked due to this contact, making it more difficult to separate during vital biological functions such as transcription and replication. The resulting structural damage causes apoptosis and cell cycle arrest, which enhances the therapeutic effectiveness of oxaliplatin. Although oxaliplatin has a primary effect on DNA, it also indirectly affects cellular proteins involved in DNA repair, namely, those involved in the NER pathway (288).

Proteins in this field include XPC (Xeroderma pigmentosum complementation group C), XPA (Xeroderma pigmentosum complementation group A), and ERCC1 (excision repair cross-complementation group 1) (289). Within the signaling pathway domain, the DDR pathway is activated by oxaliplatin-induced DNA damage. Essential proteins in this pathway, such as the ATM-encoded ataxia-telangiectasia mutated (ATM) protein and the ATR-encoded ataxia-telangiectasia and Rad3-related (ATR) protein, are critical for detecting DNA damage and coordinating biological reactions (290). These defense mechanisms include inducing cell cycle arrest, facilitating DNA repair, and encouraging apoptotic cell death if the damage is not repaired.

As an oral EGFR inhibitor, erlotinib plays a crucial role in cancer therapy by interfering with vital signaling pathways essential for cell survival and proliferation (291). Hepatic metabolism, which is primarily controlled by the cytochrome P450 enzyme system, is involved in its administration, and enzymes such as CYP3A4 and CYP3A5 play important roles (292). This metabolic pathway involves medication interactions and possible differences in drug response caused by hereditary variables. EGFR, encoded by the EGFR gene (ERBB1), is a particular protein target of erlotinib. Erlotinib inhibits EGFR, which interferes with downstream signaling cascades such as the RAS-RAF-MEK-ERK and PI3K-AKT pathways, which are required for cellular processes (293). The effectiveness of this drug is especially noteworthy in tumors with EGFR overexpression or mutations, which contribute to uncontrolled cell proliferation. Understanding erlotinib’s pharmacokinetics, molecular targets, and genetic factors is critical for customizing its usage in treating pancreatic cancer and other malignancies and enhancing therapeutic results.

### Immunotherapy

Pancreatic cancer has always been a complex disease to treat. However, immune checkpoint inhibitors have shown promise in this regard. Among these inhibitors, CTLA-4 and PD-1/PD-L1 inhibitors have attracted much interest (294). T-cell-expressed PD-1 combines with cancer cell-expressed PD-L1 to suppress the immune system (295). Monoclonal antibodies, such as nivolumab and pembrolizumab, obstruct this connection, enabling T cells to attack cancer cells efficiently (296). Similarly, CTLA-4 suppresses T-cell activation by binding with CD28 for binding affinity to antigen-presenting lymphocytes (APCs) (297). An antitumor immune response is promoted, and T-cell activation is enhanced when the CTLA-4 inhibitor ipilimumab interferes with this competition (298).

Combinations of CTLA-4 and PD-1/PD-L1 inhibitors have been studied for potential synergistic effects (299). Although these treatments have potential, they may cause immune-related side effects that require close patient observation. Biomarkers, one of which is PD-L1 expression, help patients choose and predict how well a therapy will work (300). Peptide vaccines, such as GV1001, provide a focused immunotherapeutic strategy for treating pancreatic cancer (301). These vaccines work by identifying tumor-associated antigens (TAAs), including the telomerase-derived peptide GV1001, which targets specific proteins in cancer cells (302). GV1001 has been shown in pancreatic cancer clinical trials to activate cytotoxic T cells, promoting an immune response against cancer cells that display targeted antigens (303). APCs process and deliver GV1001 to T lymphocytes, activating them to recognize and attack cancer cells. This is how the mechanism of antigen presentation works (304). GV1001 also aims to create immunological memory, guaranteeing a focused and long-lasting reaction (305). The possibility of a patient-specific design that enables modification based on unique tumor characteristics is noteworthy in terms of therapeutic concerns.

Using complete cancer cells expressing various antigens, whole-cell vaccines, such as algenpantucel-L, constitute a novel immunotherapy strategy. Algenpantucel-L is composed of irradiated pancreatic cancer cells and stimulates the immune system in a complicated way, affecting T cells, B cells, and APCs. APCs process and present a variety of antigens produced by algenpantucel-L, thereby initiating a thorough immune response. This is the mechanism of stimulation (306). Interestingly, the therapeutic considerations for whole-cell vaccines highlight their objective of concurrently targeting several antigens to elicit a more comprehensive immune response. A complex signaling cascade is used in CAR-T-cell therapy to strengthen the immune response against pancreatic cancer. T cells that have been transformed express the chimeric antigen receptor (CAR) on their surface after being given the CAR. Typically, this synthetic receptor comprises an intracellular signaling domain, a transmembrane domain, and an extracellular domain for antigen recognition (307). Costimulatory domains such as CD28 or 4-1BB (CD137) and components such as CD3ζ are often found in the intracellular signaling domain (308).

The extracellular domain of the CAR binds exclusively to the antigen on the surface of pancreatic cancer cells that express the targeted antigen, such as mesothelin (309). This binding initiates the CAR-T-cell signaling cascade. The transcription of genes linked to T-cell activation and proliferation is ultimately caused by the activation of downstream pathways by intracellular signaling domains, such as the PI3K-Akt and MAPK pathways (310). The activation of γ-chain-associated protein kinase 70 (ZAP-70) and the phosphorylation of CD3ζ are important signaling events that initiate downstream signaling cascades (311). Additional signals from costimulatory domains such as CD28 or 4-1BB improve T-cell activation, proliferation, and survival (312). When these signaling events occur, CAR-T cells produce cytotoxic chemicals, including granzymes and perforin (313). To specifically destroy pancreatic cancer cells, perforin breaks down the membrane of cancer cells, enabling granzymes to enter and cause apoptosis (314). Treatment with cytokines, including drugs such as interleukin-2 (IL-2) and interferon-alpha, is critical for treating pancreatic cancer (315).

The crucial cytokine IL-2 increases T-cell proliferation by activating the JAK-STAT signaling cascade through binding to the IL-2 receptor, which contains the IL-2Rα chain (CD25), IL-2Rβ chain (CD122), and IL-2Rγ chain (CD132) (316). This cascade improves both cell growth and effector functions. Furthermore, NK cells are activated by IL-2, which enhances their antitumor function (317). The complex signaling pathways that mediate the biological effects of IL-2 include those involving the JAK1, JAK3, and STAT proteins (318). Type I interferons, such as interferon-alpha, have anti-proliferative and immunomodulatory effects (319). When interferon-alpha binds to its receptors, such as IFNAR1 and IFNAR2, it triggers the JAK-STAT pathway, which involves STAT, JAK1, and JAK2 (320). This signaling cascade eventually strengthens the immune system’s defense against cancerous cells by controlling gene expression.

Moreover, interferon-alpha acts on several angiogenic factors to suppress angiogenesis (321). Owing to their complex mechanism of action, oncolytic viruses are a potential approach for pancreatic cancer immunotherapy. These viruses are genetically altered to increase and infect cancer cells specifically. They take advantage of the unique biology of cancer cells by focusing on hyperactive signaling pathways and weakened antiviral defenses (322).

Furthermore, viruses such as vaccinia, herpes simplex, and adenoviruses, which are often used in oncolytic virotherapy, may be modified to improve immunogenicity, tumor selectivity, and safety (323). For instance, the adenovirus E1A gene may be altered to enhance tumor selectivity. This gene encodes a protein that interacts with cellular regulators (324). Cancer cells lyse due to the infection process, releasing viral particles that spread the infection to nearby cancer cells. The release of tumor antigens during the lysis process is another benefit of this selective replication. APCs use these tumor antigens, which include proteins such as HER2/neu or carcinoembryonic antigen (CEA), to trigger an immune response (325). These cells stimulate T lymphocytes by processing and presenting antigens, mainly via the JAK-STAT signaling pathway (326).

Herpes simplex viruses may be genetically modified to contain transgenes that improve antitumor immunity, such as GM-CSF, which encodes granulocyte-macrophage colony-stimulating factor (327). Combining treatments with other modalities, such as checkpoint inhibitors such as PD-1 and PD-L1 inhibitors, is important from a clinical standpoint (328). This combination further supports long-term antitumor immunity by boosting the adaptive immune response involving CD8+ cytotoxic T cells (329). The tight ability of oncolytic viruses to limit reproduction in cancer cells is a safety concern and helps to reduce the possibility of nonspecific effects (330).

### Drugs in clinical trials for pancreatic cancer

Different medications and treatments for pancreatic cancer have been tested in clinical trials to evaluate their safety, effectiveness, and possible advantages for patients. The medications listed in **Supplementary Table S1** are quickly listed for the provided pancreatic cancer clinical trial information.

## Conclusions and future perspectives

This review seeks to provide a comprehensive analysis of PDAC, highlighting the critical molecular pathways involved, such as KRAS, Notch, and Hedgehog, and their implications for disease progression and therapy resistance. Current therapeutic strategies, including surgery, chemotherapy, and radiation, were critically examined, along with emerging treatments like immunotherapy. Despite advancements, significant challenges remain, particularly in overcoming drug resistance and the tumor’s dense stromal environment. The review also explored innovative diagnostic techniques, such as liquid biopsies, which offer a noninvasive approach for early detection, and personalized medicine, which tailors’ treatment to the patient’s genetic profile. The potential of CRISPR/Cas9 for precise genomic editing and computational intelligence for enhancing diagnostic and therapeutic efficacy was highlighted, showing promise for future advancements. The findings highlight the necessity of a multidisciplinary approach to address the complexities of pancreatic adenocarcinoma. By integrating insights from genetic, molecular, and clinical research, the review identifies key challenges and proposes future research directions. These include improving early detection methods, developing more effective therapeutic strategies, and overcoming the tumor’s immunosuppressive microenvironment.

The etiology of pancreatic cancer remains insufficiently understood, necessitating further extensive prospective studies to enhance our comprehension of the associated risk factors. Patients exhibiting a predisposition to familial PDAC could be promising candidates for screening (331). However, consensus is lacking on the optimal age, frequency, and preferred imaging techniques for screening. Conducting thorough retrospective and prospective studies that longitudinally track individuals with familial pancreatic cancer is crucial for untying disease progression and facilitating the implementation of effective screening and treatment strategies. Recognized precursors such as PanIN, IPMN, and MCN offer opportunities for early identification and intervention (332). Implementing appropriate follow-up programs based on extensive retrospective and prospective studies can ensure prompt intervention for susceptible patients and deter superfluous surgical procedures for benign lesions. These studies, conducted over extended periods, will enhance our understanding of disease processes and pinpoint determinants of the risk of these precancerous conditions, opening avenues for targeted screening in specific populations.

The advent of neoadjuvant therapy has improved survival in a few patients, yet challenges persist in identifying those who would benefit most from this approach (333). Ongoing randomized studies are needed to identify the optimal candidates for neoadjuvant therapy. The search for novel biomarkers holds promise for refining decision-making processes in an era of precision medicine, tailoring therapies to specific cases. Surgical excision, which involves vascular resection, is the cornerstone of curative intervention and offers potential benefits in achieving clear margins. However, the survival advantage associated with venous resection warrants further investigation through retrospective studies, shedding light on patient outcomes and contributing valuable insights for future guidelines (334).

Despite progress in neoadjuvant and multimodal therapies, postoperative relapses persist as a formidable challenge, necessitating innovative interventions (335). The complex interaction among neoplastic and stromal components within tumor surroundings adds complexity to the disease. Although surgery remains the primary remedial modality for initial-stage patients, patients with advanced disease require a comprehensive approach involving chemotherapeutic regimens, radiation therapy, and targeted interventions. Promising strategies for immunotherapy are hampered by immune evasion mechanisms (336).

The 5-year overall survival rate of individuals with pancreatic cancer who received FDA-approved chemotherapy and targeted therapies has increased from approximately 2% ten years ago to 11% by 2022 (337). Nevertheless, a deeper understanding of the biological intricacies inherent in PDAC subtypes has facilitated the way for more refined and targeted therapeutic strategies (338). New methodologies in clinical trial design, encompassing drug lead-in, neoadjuvant exploration of investigational agents, and the implementation of platform studies for accelerated evaluation of combinations, are driving progress. Over the subsequent decade, one might expect apparent advancements in clinical outcomes for a more extensive cohort of patients undergoing treatment with tailored combinations of therapeutic agents (339).

The novelty of this review lies in its comprehensive and integrative approach to understanding PDAC, particularly by highlighting emerging areas of research and potential therapeutic strategies that have not been extensively covered in previous literature. The review provides an updated and detailed exploration of critical molecular pathways such as KRAS, Notch, and Hedgehog, emphasizing recent discoveries and their implications for disease progression and therapy resistance. Additionally, the discussion on innovative diagnostic techniques, such as liquid biopsies, represents a significant advancement over conventional biopsy methods. Liquid biopsies offer a noninvasive means for early detection and monitoring of pancreatic cancer, providing real-time insights into tumor dynamics. The review also highlights the promise of personalized medicine, tailored to individual genetic profiles, which can optimize treatment outcomes. Furthermore, it examines the application of CRISPR/Cas9 for precise genomic editing, showcasing a cutting-edge approach to potentially correct oncogenic mutations at their source. Moreover, while previous works have discussed immunotherapy, this review provides analysis of the current challenges, particularly immune evasion mechanisms, and suggests potential strategies to overcome these hurdles. By integrating these novel insights and emerging research areas, the review not only builds upon existing knowledge but also facilitate the way for future research directions that hold promise for significantly improving the diagnosis, treatment, and overall management of PDAC.

PDAC represents a profound clinical challenge in the field of oncology, characterized by an aggressive disease course, high mortality rates, and limited therapeutic options. The prognosis for individuals with PDAC is still poor despite advancements in cancer research and treatment approaches, highlighting the dire need for a thorough comprehension of this form of cancer. A complex relationship between hereditary and environmental variables influences the development of pancreatic cancer. Elucidating the molecular pathways and signaling cascades involved in PDAC is crucial for developing novel therapeutic interventions. It provides a holistic understanding of the critical pathways, such as KRAS, Notch, Hedgehog, and Wnt/β-catenin, that drive tumor growth, metastasis, and therapeutic resistance. Even with a wide range of therapeutic options available, such as radiation therapy, chemotherapy, surgery, and targeted medicines, the overall survival rates for people with pancreatic cancer are still remarkably poor. Pancreatic cancer research is a rapidly evolving field, with numerous promising approaches on the horizon, such as immunotherapy, liquid biopsies, personalized medicine, CRISPR/Cas9 genome editing, and computational intelligence applications. PDAC is a complex disease that requires a multidisciplinary approach involving clinicians, researchers, and experts from various fields. A thorough assessment can help interdisciplinary teams work together more effectively by combining expertise from many fields, creating a common understanding, and pointing out areas where joint efforts can be made to combat this difficult illness.

## Author contributions

MM: Conceptualization, Investigation, Methodology, Resources, Validation, Writing – original draft. KA: Conceptualization, Investigation, Project administration, Resources, Writing – review & editing. MA: Formal analysis, Investigation, Methodology, Writing – original draft. SH: Conceptualization, Methodology, Supervision, Validation, Visualization, Writing – original draft. Z: Software, Funding acquisition, Methodology, Project administration, Writing – original draft. GH: Validation, Data curation, Software, Supervision, Writing – review & editing. SI: Investigation, Methodology, Validation, Writing – original draft. AS: Data curation, Investigation, Methodology, Writing – review & editing. IH: Conceptualization, Funding acquisition, Methodology, Project administration, Supervision, Writing – review & editing.
