# Supplementary material for: Investigating underlying molecular mechanisms, signaling pathways, emerging therapeutic approaches in pancreatic cancer
Source: Front Oncol. 2024 Jul 17;14:1427802. doi: 10.3389/fonc.2024.1427802 (PMC11288929; doi:10.3389/fonc.2024.1427802)
Supplement: Supplementary file 1 [file Table_1.docx]

**Table S1.** Clinical trial investigational drugs and their mechanisms of action.

| **Drug name** | **Mechanism of Action** | **Phase** | **Source** |
| --- | --- | --- | --- |
| Paclitaxel | Disrupts tubulin function | 4 | NCT05035147 |
| Gemcitabine | Blocks the activity of DNA polymerases (alpha, delta, epsilon) | 3 | NCT02993731 |
| Gemcitabine | Suppresses Ribonucleoside-diphosphate reductase RR1 | 4 | NCT04935359 |
| Capecitabine | Interferes with thymidylate synthase function | 4 | NCT04480268 |
| Nimotuzumab | Blocks erbB1 receptor activity | 4 | NCT02945267 |
| Pembrolizumab | Prevents the action of programmed cell death protein 1 | 4 | NCT03891979 |
| Dexamethasone | Binds and activates glucocorticoid receptors | 4 | NCT04025840 |
| Propofol | Enhances GABA-A receptor function as a positive allosteric modulator | 3 | NCT03434678 |
| Sorafenib | Suppresses activity of the VEGFR | 3 | NCT00541021 |
| Rocuronium | Acts as an antagonist to muscle-type nicotinic acetylcholine receptors | 3 | NCT03434678 |
| Sorafenib | Suppresses the activity of the tyrosine-protein kinase receptor RET | 3 | NCT00541021 |
| Ruxolitinib | Blocks the function of tyrosine-protein kinase JAK1 | 3 | NCT02117479 |
| Octreotide | Activates somatostatin receptors | 3 | NCT02457156 |
| Camrelizumab | Counteracts the action of programmed cell death protein 1 | 3 | NCT04674956 |
| Irinotecan | Suppresses the function of DNA topoisomerase I | 3 | NCT03665441 |
| Pamrevlumab | Blocks the activity of connective tissue growth factor | 3 | NCT03941093 |
| Sargramostim | Activates the granulocyte-macrophage colony-stimulating factor receptor | 3 | NCT00425360 |
| Celecoxib | Inhibits the activity of cyclooxygenase-2 | 3 | NCT00486460 |
| Masitinib | Suppresses the function of stem cell growth factor receptor | 3 | NCT03766295 |
| Bevacizumab | Suppresses the activity of vascular endothelial growth factor A | 3 | NCT00088894 |
| Masitinib | Suppresses the function of PGFR | 3 | NCT00789633,NCT03766295 |
| Relacorilant | Counters the action of glucocorticoid receptors | 3 | NCT04329949 |
| Erfonrilimab | Blocks cytotoxic T-lymphocyte protein 4 | 3 | NCT05149326 |
| Tegafur | Interferes with thymidylate synthase function | 3 | NCT00498225 |
| Sunitinib | Suppresses the activity of macrophage colony-stimulating factor receptor | 3 | NCT00428597 |
| Olaparib | Suppresses poly [ADP-ribose] polymerase 1, 2, and 3 | 3 | NCT02184195 |
| Pasireotide | Activates somatostatin receptors 1 | 3 | NCT00994110 |
| Ruxolitinib | Suppresses the function of tyrosine-protein kinase JAK2 | 3 | NCT02119663,NCT02117479 |
| Exatecan | Suppresses the function of DNA topoisomerase I | 3 | NCT00023972 |
| Bupivacaine | Blocks the sodium channel protein type IV alpha subunit | 3 | NCT03434678 |
| Methylnaltrexone bromide | Acts as an antagonist to the mu opioid receptor | 3 | NCT04151719 |
| Gimeracil | Suppresses the activity of dihydropyrimidine dehydrogenase | 3 | NCT00498225 |
| Dalteparin sodium | Stimulates the activity of antithrombin-III | 3 | NCT00031837 |
| Pegilodecakin | Activates the IL-10 receptor | 3 | NCT02923921 |
| Spartalizumab | Counters the action of programmed cell death protein 1 | 3 | NCT04229004 |
| Ibrutinib | Suppresses the function of tyrosine-protein kinase BTK | 3 | NCT02436668 |
| Dipyridamole | Suppresses the activity of cyclic phosphodiesterase (3',5') | 2 | NCT00003018 |
| Denileukin diftitox | Blocks the binding of interleukin-2 to its receptor | 2 | NCT00726037 |
| Niraparib | Suppresses poly [ADP-ribose] polymerase 2 | 2 | NCT04409002,NCT03553004 |
| Regorafenib | Suppresses the function of stem cell growth factor receptor | 2 | NCT02383433 |
| Losartan | Acts as an antagonist to the type-1 angiotensin II receptor | 2 | NCT04539808 |
| Valproic acid | Suppresses the activity of succinate semialdehyde dehydrogenase | 2 | NCT01333631 |
| Selumetinib sulfate | Suppresses MEK1/2, a dual specificity mitogen-activated protein kinase kinase | 2 | NCT03040986 |
| Indusatumab vedotin | Disrupts tubulin function | 2 | NCT02202785 |
| Vandetanib | Suppresses the activity of ephrin receptors | 2 | NCT01601808 |
| Nivolumab | Suppresses the action of programmed cell death protein 1 | 2 | NCT03080974 |
| Pazopanib | Suppresses the activity of the VEGFR | 2 | NCT01080248 |
| Docetaxel | Disrupts tubulin function | 2 | NCT00112697,NCT00996333,NCT00238199,NCT01459614,NCT00966706,NCT00137761,NCT00004884,NCT00042939,NCT00290693,NCT00012220,NCT01186731,NCT01905150,NCT00003810,NCT00761241,NCT00882310,NCT00869258 |
| Arsenic trioxide | Suppresses the activity of thioredoxin reductase 1 | 2 | NCT00053222 |
| Metformin | Suppresses the function of mitochondrial complex I (NADH dehydrogenase) | 2 | NCT01167738 |
| Panobinostat | Suppresses the activity of histone deacetylase | 2 | NCT01056601 |
| Dasatinib | Suppresses the activity of SRC, a tyrosine kinase | 2 | NCT01652976,NCT01395017 |
| Elraglusib | Suppresses the activity of glycogen synthase kinase-3 beta | 2 | NCT05239182 |
| Rg-4733 | Blocks the activity of gamma-secretase | 2 | NCT01232829 |
| Doxycycline | Suppresses the activity of matrix metalloproteinase 13 | 2 | NCT02775695 |
| Dolastatin-10 | Disrupts tubulin function | 2 | NCT00003677 |
| Ganitumab | Acts as an antagonist to the insulin-like growth factor I receptor | 2 | NCT01318642 |
| Phenytoin | Blocks the sodium channel alpha subunit. | 2 | NCT03512756 |
| Bethanechol | Activates muscarinic acetylcholine receptor M2 | 2 | NCT05241249 |
| Famitinib | Suppresses the function of PGFR | 2 | NCT04814485 |
| Atezolizumab | Suppresses the action of programmed cell death 1 ligand 1 | 2 | NCT04820179 |
| Pertuzumab | Suppresses the activity of receptor protein-tyrosine kinase erbB-2 | 2 | NCT01108458 |
| Trametinib | Suppresses MEK1/2, a dual specificity MAPK | 2 | NCT02428270 |
| Etoposide | Suppresses DNA topoisomerase II | 2 | NCT00202800 |
| Fruquintinib | Suppresses the activity of the VEGFR | 2 | NCT05168527 |
| Interferon alfa-2b | Activates the interferon alpha/beta receptor | 2 | NCT00059826 |
| Enmd-981693 | Suppresses the activity of SRC, a tyrosine kinase. | 2 | NCT00568646 |
| Ixabepilone | Disrupts tubulin function | 2 | NCT00383149,NCT00016965 |
| Urelumab | Activates TNF receptor superfamily member 9. | 2 | NCT02451982 |
| Denileukin diftitox | Suppresses the function of elongation factor 2 | 2 | NCT00726037 |
| Tislelizumab | Suppresses the action of programmed cell death protein 1 | 2 | NCT05634564,NCT04902261 |
| Ucn-01 | Suppresses the activity of protein kinase C | 2 | NCT00045747 |
| Vismodegib | Blocks the activity of smoothened homolog | 2 | NCT01096732 |
| Digoxin | Suppresses the function of sodium/potassium-transporting ATPase | 2 | NCT04141995 |
| Azacitidine | Suppresses the activity of DNA (cytosine-5)-methyltransferase 3A | 2 | NCT01845805 |
| Gsk-2256098 | Suppresses the activity of focal adhesion kinase 1 | 2 | NCT02428270 |
| Sx-682 | Blocks interleukin-8 receptors, CXCR1/CXCR2 | 2 | NCT05604560 |
| Acalabrutinib | Suppresses the function of tyrosine-protein kinase BTK | 2 | NCT02362048 |
| Ucn-01 | Suppresses the activity of cyclin-dependent kinase 6 | 2 | NCT00045747 |
| Abemaciclib | Suppresses the activity of cyclin-dependent kinase 4 | 2 | NCT02981342 |
| Tacedinaline | Suppresses the activity of histone deacetylase | 2 | NCT00004861 |
| Binimetinib | Suppresses MEK1/2, a bifunctional MAPK | 2 | NCT04390243 |
| Samotolisib | Suppresses the activity of PI3-kinase class I | 2 | NCT02981342 |
| Anamorelin | Activates the ghrelin receptor | 2 | NCT04844970 |
| Pioglitazone | Activates peroxisome proliferator-activated receptor gamma | 2 | NCT01838317 |
| Temsirolimus | Suppresses FK506-binding protein 1A | 2 | NCT00075647 |
| Lenalidomide | Suppresses CRL4(CRBN) E3 ubiquitin ligase | 2 | NCT00837031 |
| Cabozantinib | Suppresses the activity of hepatocyte growth factor receptor | 2 | NCT03213626 |
| Veliparib | Suppresses poly [ADP-ribose] polymerase 1, 2, and 3 | 2 | NCT02890355,NCT01585805 |
| Raltitrexed | Interferes with thymidylate synthase function | 2 | NCT04581876 |
| Tanespimycin | Suppresses the activity of heat shock protein HSP90 | 2 | NCT00577889 |
| Nirogacestat | Blocks the activity of gamma-secretase | 2 | NCT02109445 |
| Mk-4721 | Suppresses prostate stem cell antigen | 2 | NCT01608711,NCT00902291 |
| Epacadostat | Suppresses the activity of indoleamine 2,3-dioxygenase | 2 | NCT03006302 |
| Metformin | Suppresses the activity of mitochondrial glycerol-3-phosphate dehydrogenase | 2 | NCT02978547 |
| Tipifarnib | Suppresses the activity of protein farnesyltransferase | 2 | NCT00005843,NCT00005832 |
| Anlotinib | Suppresses the function of stem cell growth factor receptor | 2 | NCT04718701 |
| Triapine | Suppresses the activity of ribonucleotide reductase | 2 | NCT00064051,NCT00078975 |
| Alvocidib | Suppresses the activity of cyclin-dependent kinase 7 | 2 | NCT00331682 |
| Tipiracil | Suppresses the activity of thymidine phosphorylase | 2 | NCT02921737 |
| Pemetrexed | Interferes with thymidylate synthase function | 2 | NCT00864513 |
| Cetuximab | Suppresses the activity of epidermal growth factor receptor erbB1 | 2 | NCT00871169,NCT00225784,NCT00305760,NCT00305877,NCT00599833,NCT00338039,NCT00536614,NCT00408564,NCT00383149,NCT00042939 |
| Imatinib | Suppresses the function of stem cell growth factor receptor | 2 | NCT00161213 |
| Pci-27483 | Suppresses the activity of coagulation factor VII | 2 | NCT01020006 |
| Doxorubicin | Suppresses the function of DNA topoisomerase II alpha | 2 | NCT00609765,NCT00426127 |
| Zolbetuximab | Binds to claudin-18 | 2 | NCT03816163 |
| Cdx-301 | Activates tyrosine-protein kinase receptor FLT3 | 2 | NCT04536077 |
| Lapatinib | Suppresses the activity of epidermal growth factor receptor erbB1 | 2 | NCT00881621 |
| Filgrastim | Activates granulocyte colony-stimulating factor receptor | 2 | NCT03042780 |
| Cdx-1140 | Activates tumor necrosis factor receptor superfamily member 5 | 2 | NCT04536077 |
| Motixafortide | Blocks C-X-C chemokine receptor type 4 | 2 | NCT02826486 |
| Rintatolimod | Activates toll-like receptor 3 | 2 | NCT05494697 |
| Escitalopram | Suppresses the activity of the serotonin transporter | 2 | NCT05289830 |
| Ipilimumab | Blocks cytotoxic T-lymphocyte protein 4 | 2 | NCT05014776,NCT05116917 |
| Pazopanib | Suppresses the function of stem cell growth factor receptor | 2 | NCT01080248 |
| Paricalcitol | Activates the vitamin D receptor | 2 | NCT04524702 |
| Sintilimab | Counters the action of programmed cell death protein 1 | 2 | NCT05562297 |
| Disulfiram | Suppresses the activity of aldehyde dehydrogenase | 2 | NCT03714555 |
| Everolimus | Suppresses FK506-binding protein 1A | 2 | NCT00409292 |
| Cadonilimab | Suppresses the action of programmed cell death protein 1 | 2 | NCT05859750 |
| Saracatinib | Suppresses the function of tyrosine-protein kinase ABL | 2 | NCT00735917 |
| Zoledronic acid | Suppresses farnesyl diphosphate synthase | 2 | NCT03073785 |
| Anakinra | Acts as an antagonist to interleukin-1 receptor | 2 | NCT04926467 |
| Ss1(dsfv)-pe38 | Binds to mesothelin | 1 | NCT00006981,NCT00066651 |
| Lenvatinib | Suppresses the activity of vascular endothelial growth factor receptor | 1 | NCT05303090 |
| Umbralisib | Suppresses the activity of casein kinase I epsilon | 1 | NCT02574663 |
| Birabresib | Suppresses bromodomain and extra-terminal motif (BET) | 1 | NCT02259114 |
| Sotigalimab | Activates TNF receptor superfamily member 5 | 1 | NCT05419479 |
| Cobimetinib | Suppresses MEK1/2, a dual specificity mitogen-activated protein kinase | 1 | NCT05034627,NCT03193190 |
| Enoxaparin sodium | Activates antithrombin-III | 1 | NCT01945879 |
| Tiragolumab | Suppresses T-cell immunoreceptor with Ig and ITIM domains | 1 | NCT03193190 |
| Zimberelimab | Suppresses the action of programmed cell death protein 1 | 1 | NCT05419479 |
| Surufatinib | Suppresses the activity of the VEGFR | 1 | NCT05832892 |
| Palbociclib | Suppresses the activity of CDK6/cyclin D1 | 1 | NCT05039177 |
| Fulvestrant | Acts as an antagonist to the estrogen receptor | 1 | NCT04247126 |
| Romidepsin | Suppresses the activity of histone deacetylase | 1 | NCT04257448 |
| Vantictumab | Acts as an antagonist to frizzled-8 | 1 | NCT02005315 |
| Sonidegib | Blocks the activity of smoothened homolog | 1 | NCT01485744 |
| Bintrafusp alfa | Binds to TGF β | 1 | NCT03451773 |
| Tadalafil | Suppresses activity of phosphodiesterase 5A | 1 | NCT01342224 |
| Durvalumab | Suppresses the action of programmed cell death 1 ligand 1 | 1 | NCT02583477 |
